# Effect of Nanoparticle Rigidity on the Interaction of Stromal Membrane Particles with Leukemia Cells

**DOI:** 10.1002/adhm.202500667

**Published:** 2025-06-08

**Authors:** Sander de Weerd, Xinyu Ma, Zahra Zohali, Lieve L. Oudejans, Emmanouil Kyrloglou, Marc. C. A. Stuart, Wouter H. Roos, Jan Jacob Schuringa, Anna Salvati

**Affiliations:** ^1^ Nanomedicine and Drug Targeting Groningen Research Institute of Pharmacy University of Groningen A. Deusinglaan 1 Groningen 9713 AV The Netherlands; ^2^ Molecular Biophysics Zernike Institute for Advanced Materials University of Groningen Nijenborgh 3 Groningen 9747 AG The Netherlands; ^3^ Department of Experimental Hematology University Medical Center Groningen University of Groningen Groningen 9713 GZ The Netherlands; ^4^ Electron Microscopy Groningen Biomolecular Sciences and Biotechnology Institute University of Groningen Nijenborgh 7 Groningen 9747 AG The Netherlands

**Keywords:** cell membrane nanoparticles, leukemia, lipid‐coated nanoparticles, nanoparticle mechanical properties, nanoparticle targeting

## Abstract

In acute myeloid leukemia (AML), disease relapse is often observed because of therapy‐resistant leukemic stem cells that re‐initiate the disease. Leukemic stem cells can tightly associate with mesenchymal stromal cells inside the bone marrow, which is considered to further drive drug resistance. Here, the cell membrane of bone marrow stromal cells is used to prepare cell membrane nanoparticles and study their interactions with AML cells. Cell membrane liposomes (CM‐Liposomes) of different charge are prepared and either used directly, or after deposition on silica cores to modulate nanoparticle mechanical properties. Nanoparticle size, zeta potential and coating efficiency are analyzed by dynamic light scattering (DLS) and cryo electron microscopy (Cryo‐EM) imaging. Atomic force microscopy (AFM) is used to characterize the mechanical properties of CM‐Liposomes and confirm bilayer deposition on silica cores. Finally, uptake by leukemic cells is determined. No difference in uptake is found between soft CM‐Liposomes and liposomes of the same composition without membrane components. Instead, after deposition on a rigid core, uptake is higher for the cell membrane particles. Preliminary results on primary cells from leukemia patients confirm this observation. These results show that nanoparticle rigidity strongly affects the interaction between cell membrane nanoparticles and the targeted cells.

## Graphical abstract

Cell membrane nanoparticles made with stromal cells are prepared and used to target leukemia cells. Deposition of the cell membrane onto rigid silica cores leads to increased uptake both in leukemia cell lines and primary cells from patients. These results show that nanoparticle rigidity affects the interaction between cell membrane nanoparticles and the target cells. (TOC created in BioRender, de weerd, S. (2024) https://BioRender.com/t25s047).

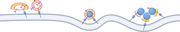

## 1 Introduction

Cell membrane liposomes (CM‐Liposomes) and cell membrane‐coated nanoparticles have attracted increasing interest for their potential use as drug carriers. By displaying cell membrane components on the surface of nanoparticles, naturally occurring cellular interactions or functions can be exploited for drug delivery, vaccination or treatment.^[^ ^1^, ^2^, ^3^, ^4^, ^5^ ^]^ For instance, cell membrane camouflaged nanoparticles have been shown to enable longer circulation time.^[^ ^2^, ^6^ ^]^ Additionally, cancer cell membrane nanoparticles take advantage of the interactions between cancer cells to achieve so called “homotypic targeting”.^[^ ^5^, ^7^, ^8^, ^9^ ^]^ Expressing cancer antigens on a cell membrane nanoparticle made from cancer cells provides additional opportunities for vaccination.^[^ ^5^ ^]^ Recently, cmembranes have even been engineered with artificial SpyCatcher receptors, that can form bonds with any protein of interest offering unprecedented functionality.^[^ ^10^, ^11^ ^]^ The idea behind these applications is that membrane proteins at the nanoparticle surface interact specifically with the targeted cells. However, the core principles of how these membrane mimics manage (or fail) to facilitate interaction with receptors on the interacting cells are still poorly understood.

**Figure 1:**
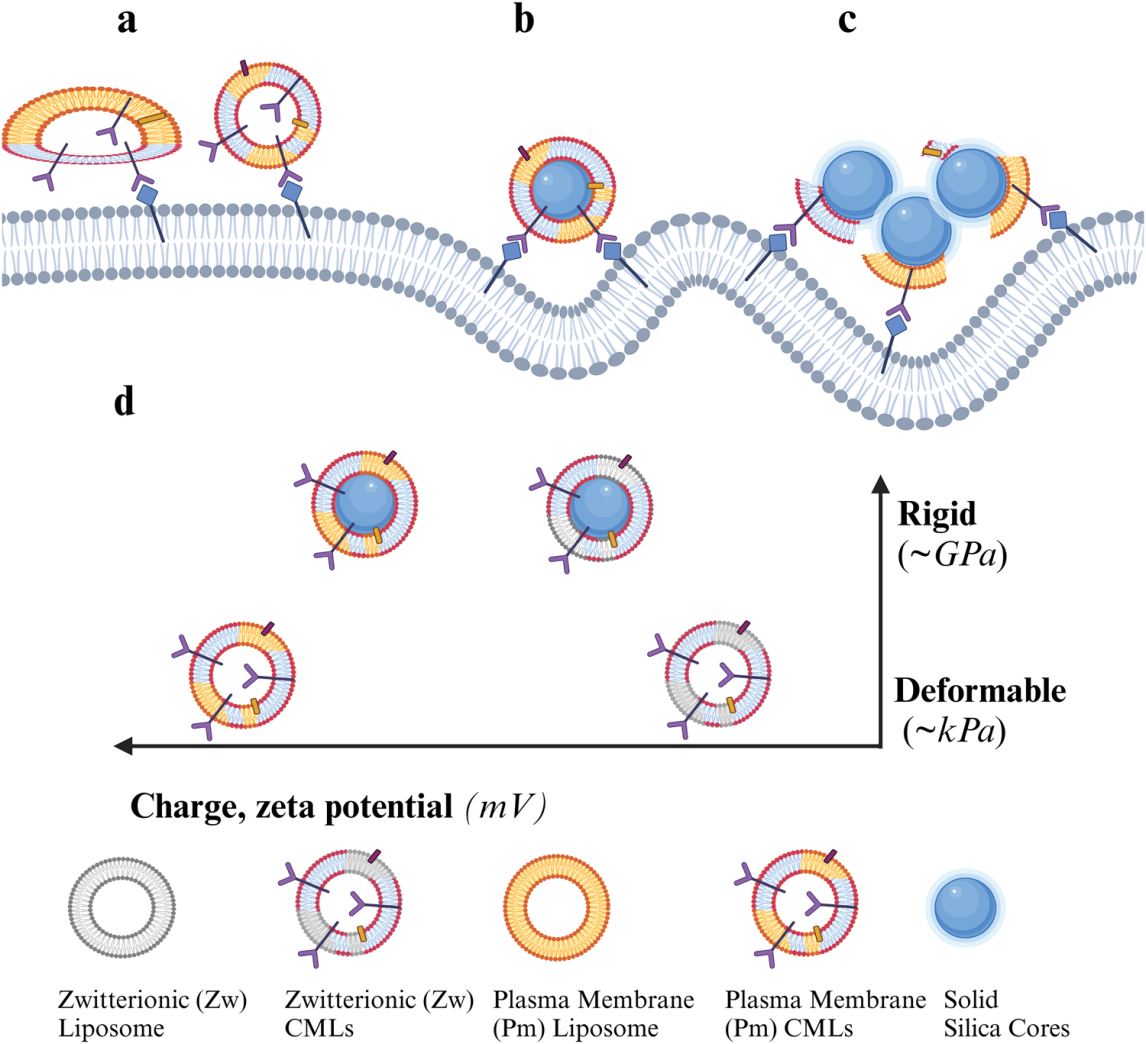
Schematic representation of the hypotheses tested in this study. The aim of this work was to study how nanoparticle rigidity and charge influence the interaction of cell membrane nanoparticles with the target cells: a) Softer and deformable CM‐Liposomes (CMLs) may not be able to bend the cell membrane and have low contact area.^[^ ^22^ ^]^ b) Rigid membrane coated nanoparticles may press into the target cell membrane and bend it.^[^ ^22^, ^24^ ^]^ c) Agglomerates of partially coated rigid membrane nanoparticles may form and may press into the cell membrane, increasing the contact area.^[^ ^25^ ^]^ d) Schematic overview of soft and rigid model nanoparticles used in this study organized by charge (zeta potential, x‐axis) and mechanical properties (rigidity, y‐axis). Created in BioRender, de Weerd, S. (2024) https://BioRender.com/k44t679.

It is generally known that uptake of nanoparticles is influenced heavily by nanoparticle properties, such as their size, shape, and charge.^[^ ^12^, ^13^, ^14^, ^15^ ^]^ Recently, nanoparticle mechanical properties have also been indicated to affect biodistribution and cellular uptake.^[^ ^16^, ^17^, ^18^ ^]^ However, conflicting results have been reported on whether uptake is favored for softer or more rigid nanoparticles,^[^ ^19^, ^20^ ^]^ also depending on the type of nanomaterials and their rigidity range,^[^ ^20^ ^]^ as well as the cells used for the study.^[^ ^20^ ^]^ For example, rigid lipid‐coated silica (LCS) showed higher uptake compared with soft liposomes of same composition.^[^ ^21^ ^]^ One of the explanations for the higher uptake observed in many cases for more rigid materials is that rigid particles are more easily enveloped by the membrane and ‐ as a consequence ‐ by their receptors, increasing interaction, thus facilitating their internalization.^[^ ^22^ ^]^ A lot of the work so far has used synthetic (lipid) particles and layer‐by‐layer particles to prepare nanoparticles of different mechanical properties, due to their ease of manufacturing, and easy control of rigidity by varying cross‐linking density.^[^ ^20^ ^]^ Instead, in the context of biological cell membrane nanoparticles less is known on how the mechanical properties modulate the interactions between the membrane proteins on the nanoparticle and those on the interacting cells. Here, we will argue that similar mechanisms may be present, where more rigid membrane nanoparticles may press into the cell membrane of the targeted cells (**Figure** **1**a,b),^[^ ^22^, ^23^ ^]^ leading to a larger contact area, and in this way allowing for more proteins on the cell membrane nanoparticle to (specifically) interact with the target membrane and vice versa.^[^ ^24^ ^]^ In addition, some studies have suggested that partially covered cell membrane nanoparticles can dynamically form small agglomerates, allowing nanoparticles to interact with multiple receptors on the membrane (Figure 1c).^[^ ^25^ ^]^ According to this hypothesis, the increased contact area upon partial agglomeration on the cell surface aids interaction with multiple cell receptors and – in this way – promotes efficient internalization of the membrane nanoparticles.^[^ ^25^ ^]^

Within this context, the aim of this work was (I) to determine whether CM‐Liposomes made with the membrane of bone marrow stromal cells could be used to promote interaction with leukemia cancer (stem) cells and (II) to modulate CM‐Liposome rigidity and charge to assess their effects on the resulting interactions with the targeted cells.

Leukemia cancer cells are known to strongly interact with stromal cells in the bone marrow and the bone marrow niche heavily influences the pathophysiology of leukemia.^[^ ^26^, ^27^, ^28^, ^29^, ^30^, ^31^, ^32^ ^]^ Consistent with this, in vitro studies showed that leukemia cancer cells strongly interact with MS5 mouse stromal cells and are supported by them in culture.^[^ ^33^, ^34^, ^35^, ^36^, ^37^ ^]^ Furthermore, leukemic CM‐Liposomes or coated particles have been used to target and treat the bone marrow niche,^[^ ^9^, ^38^, ^39^ ^]^ and haematopoietic stem cells or nitrogen‐treated leukemia cells have been used to deliver drugs to the niche as well.^[^ ^40^, ^41^ ^]^ Based on these observations, rather than common approaches in cell membrane nanotechnology, where CM‐Liposomes made with the membrane of the targeted cells are used to exploit “homotypic targeting”, we were interested to study whether and how CM‐Liposomes derived from interacting stromal cells could be used to improve nanoparticle uptake by leukemia cells and leukemia stem cells.^[^ ^42^, ^43^ ^]^

To this end, zwitterionic liposomes and negatively charged liposomes made from a mix of lipids resembling cell membrane composition were prepared (Figure 1).^[^ ^44^, ^45^ ^]^ Zwitterionic lipids are used frequently because of their low unspecific binding to membranes.^[^ ^46^ ^]^ CM‐Liposomes were generated by adding MS5 bone marrow stromal cell membrane to the two types of liposomes and their mechanical properties and accumulation in leukemia cell lines were investigated. The same liposomes and CM‐Liposomes were also deposited on silica nanoparticles to test the effect of the increased rigidity upon deposition on a hard inorganic core on the resulting interactions with the leukemia cells (Figure 1). The size, charge, coating efficiency, and mechanical properties of the different liposomes and CM‐Liposomes were characterized using Dynamic Light Scattering (DLS), Atomic Force Microscopy (AFM), and Cryo‐Electron Microscopy (Cryo‐EM) imaging. Using flow cytometry to quantify uptake, the affinity of each formulation toward relevant leukemia cell lines (K562, THP1, MOLM13) was investigated. Finally, for the functional compositions preliminary experiments on human patients primary leukemic blasts and stem cells were performed, in order to confirm the observations in more relevant cells. This allowed us to investigate how nanoparticle mechanical properties affect the interaction of cell membrane nanoparticles with cells.

## 2 Results

### 2.1 Soft Liposomes and CM‐Liposomes Show Equal Nanoparticle Accumulation and Uptake Mechanism

MS5 bone marrow stromal cells were chosen to extract cell membranes for CM‐Liposome preparation and target leukemia cells because of the leukemia cells’ natural affinity for bone marrow stroma.^[^ ^26^, ^27^, ^29^, ^32^, ^34^, ^35^, ^47^ ^]^ The murine MS5 bone marrow stromal line has long been used to study the biology of human hematopoietic stem cells (HSCs) given its capacity to support long‐term in vitro growth of HSCs, and also to quantify the number of human HSCs in long‐term culture initiating cell (LTC‐IC) assays^[^ ^48^, ^49^, ^50^, ^51^, ^52^, ^53^, ^54^, ^55^ ^]^ and various others). Additionally, MS5 stromal cells have been extensively used for acute myeloid leukemia (AML) research as they also support long‐term in vitro cultures of primary patient‐derived AML blasts^[^ ^35^, ^36^, ^49^, ^50^, ^56^ ^]^ and various others). Given the vast body of literature on these stromal cells as support for human normal and malignant HSCs, and also because the interaction between AML leukemic stem cells and MS5 stromal cells appeared to be critically important to sustain in vitro self‐renewal, we opted to use these cells for the generation of membrane‐coated nanoparticles in our current study. Nevertheless, human‐specific adhesion factors that would not be expressed on murine MS5 stromal cells may be missed. Hence, for future studies, it will be of interest to also include human mesenchymal cells to develop this concept further for drug delivery applications.

**Figure 2:**
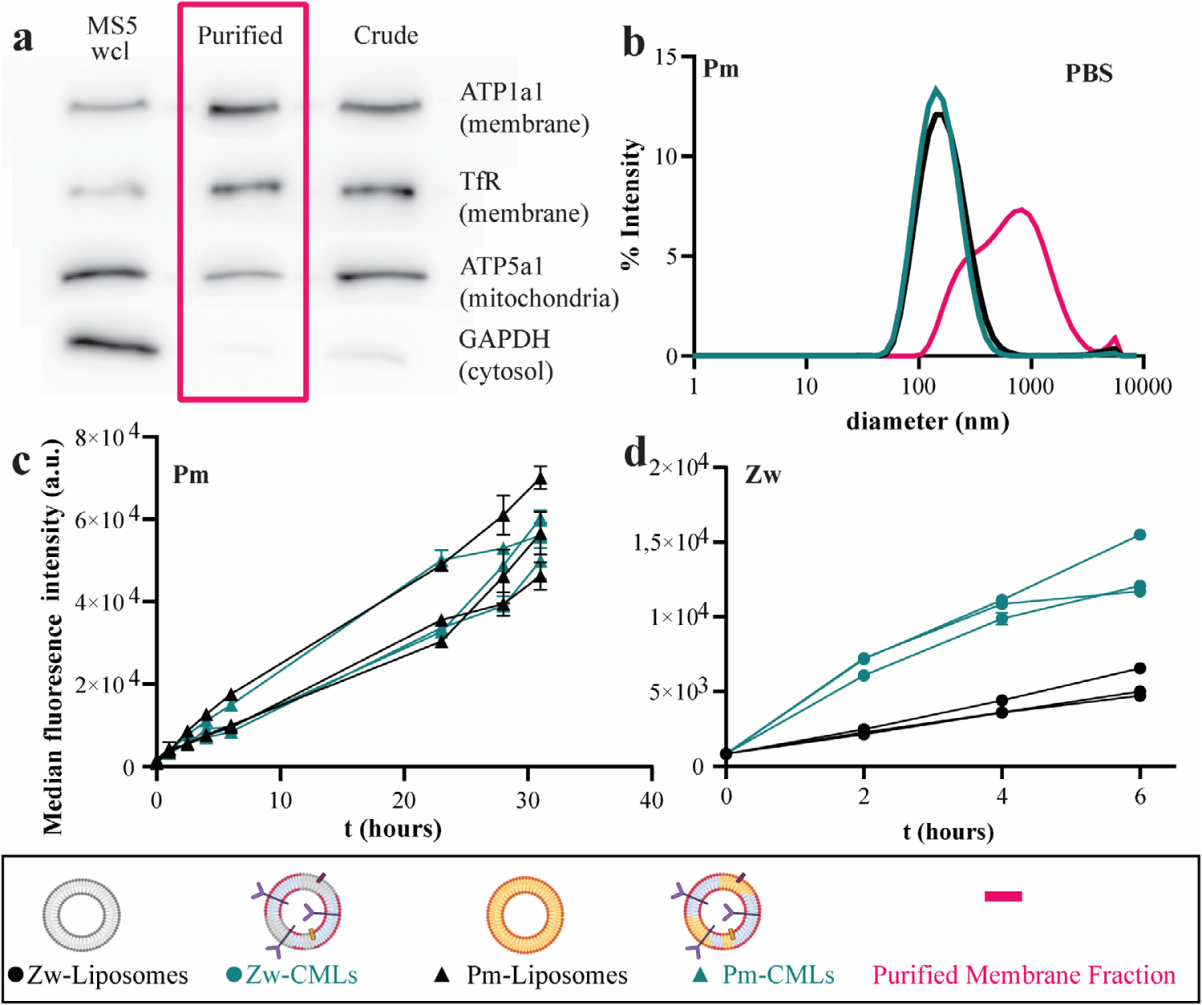
Characterization and cell uptake of liposomes and MS5‐CM‐Liposomes. a) Western blot of a whole cell RIPA lysate (MS5 wcl), crude membrane fraction (Crude), and purified membrane fraction (Purified, magenta) obtained with a cell membrane purification protocol optimized previously (*n* = 4, one representative blot is shown).^[^ ^4^, ^9^ ^]^ Membranes were stained with markers for the plasma membrane, mitochondria, and cytosol in order to determine the composition of the different fractions (see Experimental Section for details). b) Size distribution of Pm‐Liposomes (black, *n* = 10, each measured in triplicate), Pm‐CM‐Liposomes (Pm‐CMLs, green, *n* = 10, each measured in triplicate) and purified membrane vesicles (magenta, one batch shown, measured in triplicate) in PBS (25 µg mL^−1^ lipid for liposomes and CM‐Liposomes and ≈0.10 mg mL^−1^ protein for membrane vesicles). c) Average median fluorescence intensity of K562 cells incubated with 10 µg mL^−1^ Pm‐Liposomes (black‐triangles, mean ± SD, *n* = 3) and Pm‐CM‐Liposomes (green‐triangles, mean ± SD, *n* = 2) in 10% FBS over time, and to d) 50 µg mL^−1^ Zw‐Liposomes (black‐circles) and Zw‐CM‐Liposomes (Zw‐CMLs, green‐triangles). Error bars are always included but too small to see in panel d. Figure legends created with Biorender.com.

First, MS5 were lysed using nitrogen cavitation, and plasma membrane vesicles of high purity were obtained using differential centrifugation and sucrose gradient fractionation, following a protocol previously developed in our lab (**Figure** **2**a).^[^ ^4^, ^9^ ^]^ Next, liposomes and CM‐Liposomes were generated using various plasma membrane extracts to verify the robustness of the membrane extraction and particle preparation methods and showed high reproducibility (Figure S1, Supporting Information). A zwitterionic (Zw) lipid mixture and a mixture which resembles a plasma membrane (Pm) were chosen to prepare particles (see Experimental Section for details). In a previous work, the zwitterionic mixture was used for deposition of a bilayer on a silica core, showing very good coverage.^[^ ^21^ ^]^ Upon inclusion of membrane vesicles in the Zw mixture, the Z‐average diameter slightly decreased and the zeta potential became slightly more negative, consistent with inclusion of negatively charged membrane lipids, glycolipids, and proteins in the zwitterionic bilayer (Figure S1a–c, Supporting Information). The Pm mixture, instead, was optimized to mimic cell membrane composition,^[^ ^25^, ^44^, ^57^ ^]^ and was used in a previous work for the preparation of CM‐Liposomes made with the membrane of K562 leukemia cells.^[^ ^9^ ^]^ In water, slightly smaller Pm liposomes were obtained upon the addition of MS5 membrane vesicles, with a less negative zeta potential (Figure S1, Supporting Information and overview of results in **Table** **1**). Pm‐CM‐Liposomes had a slightly smaller size and lower polydispersity index (PDI), but similar zeta potential in PBS in comparison to the Pm‐liposomes without membrane components (Figure S1d–f, Supporting Information). All compositions were stable in medium with 10% serum, as used for cell culture, and were actively internalized (Figure S2a–d, Supporting Information). Contrary to what was previously observed for Pm‐CM‐Liposomes made from the membrane of K562 themselves,^[^ ^9^ ^]^ when using MS5 membrane for nanoparticle preparation, no strong differences in uptake could be observed for the Pm‐Liposomes and Pm‐CM‐Liposomes in both K562 (Figure 2c) and in MOLM13 leukemia cells (Figure S2e, Supporting Information). Even when varying the cholesterol percentage from 0, to 10 and 33 mol% to partially tune bilayer rigidity,^[^ ^58^ ^]^ only small differences in uptake could be observed (Figure S3, Supporting Information). A small increase in uptake could be observed for the Zw‐CM‐Liposomes in respect to Zw‐liposomes in K562 (Figure 2d, ≈2‐3 fold) and THP‐1 (Figure S2f, Supporting Information, ≈1‐2 fold) cells. However, this is possibly explained by the more negative zeta potential of the Zw‐CM‐Liposomes (Figure 2e) in comparison to the Zw‐liposomes, rather than specific interactions mediated by the added cell membrane components. To test whether, despite the similar uptake efficiency, Pm‐CM‐Liposomes and Pm‐Liposomes were internalized via different pathways, a panel of transport inhibitors was used to characterize the mechanisms involved.^[^ ^59^ ^]^ No clear differences in uptake mechanisms could be determined and for both nanoparticles clathrin‐mediated endocytosis and macropinocytosis seemed to be involved (Figure S4, Supporting Information).

**Table 1:** Zeta‐potential, size (Z‐average diameter), and polydispersity index (PDI) obtained by cumulant analysis of DLS data in low ionic strength water of liposomes and CM‐Liposomes used in this work (Mean ± SD, *n* = 4 batches of particles, each measured in triplicate).

Particle	Zeta [mV]	Size [Z‐average]	PDI
Zw‐Liposomes	−10 ± 8	179 ± 30	0.13 ± 0.03
Zw‐CM‐Liposomes	−12 ± 1	165 ± 19	0.19 ± 0.06
Pm‐Liposomes	−59 ± 2	165 ± 14	0.18 ± 0.04
Pm‐CM‐Liposomes	−52 ± 2	131 ± 8	0.19 ± 0.03

### 2.2 Liposomes and MS5 CM‐Liposomes Have Similar Rigidity and Are Both Soft

**Figure 3:**
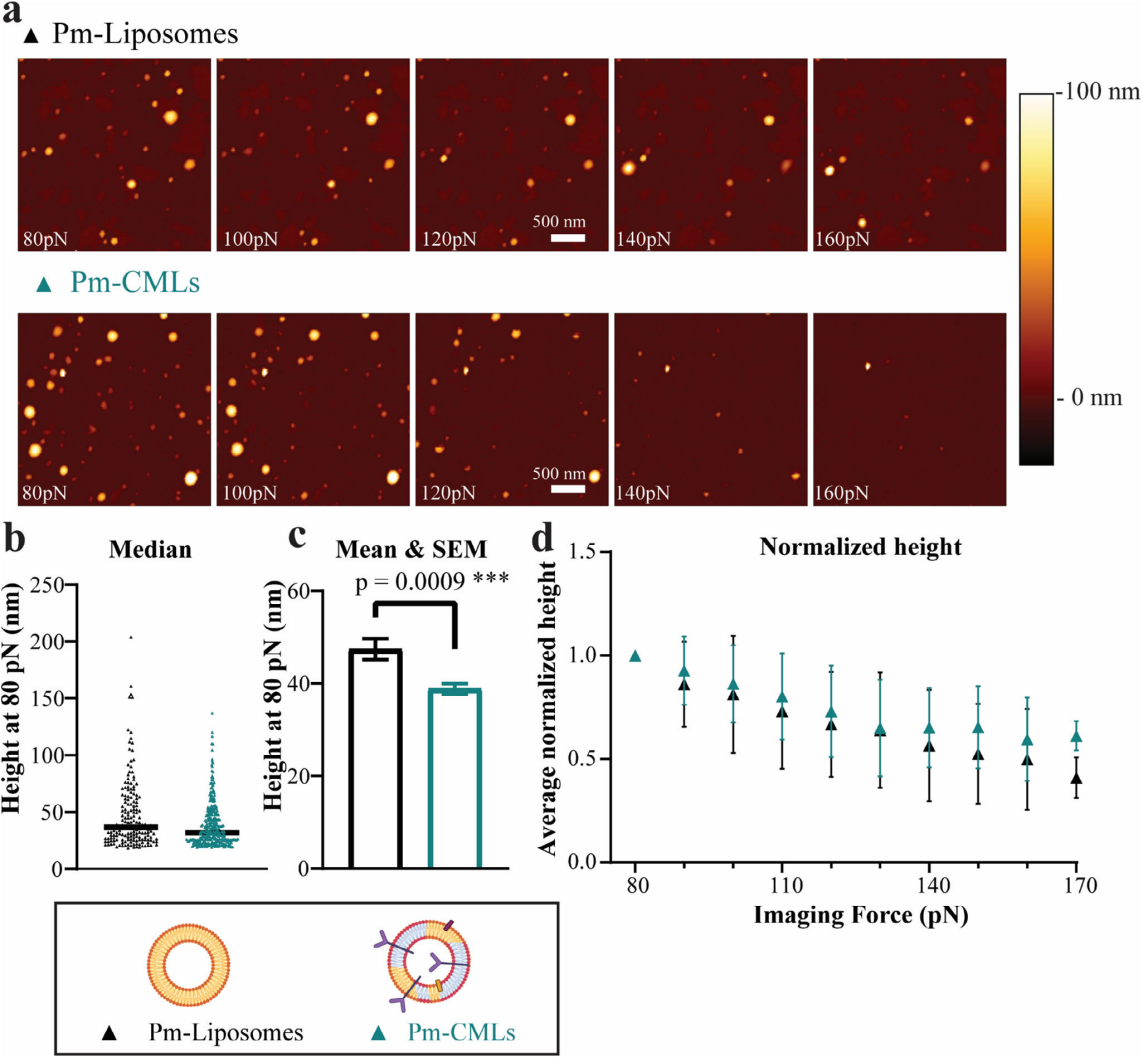
Mechanical properties of liposomes and MS5‐CM‐Liposomes, as characterized by AFM imaging. a) Examples of AFM images of Pm‐Liposome and Pm‐CM‐Liposome (Pm‐CMLs). b,c) Heights of individual Pm‐Liposomes (*n* = 179, black‐triangles) and Pm‐CM‐Liposomes (*n* = 315, green‐triangles) in PBS from a representative batch at 80 pN, together with the calculated median (b), and the calculated mean and SEM (c). A Welch's‐t‐test was used to compare the mean values in panel c (*p* = 0.0009). d) Normalized height data for both nanoparticles at increasing imaging forces (Average ± SD). The comparable change in height suggests that both particles have similar mechanical properties and are equally soft. Figure legends created with Biorender.com.

Having observed that Pm‐CM‐Liposomes and Pm‐Liposomes do not show much differences in uptake in leukemia model cell lines, their mechanical properties were compared. The negatively charged Pm‐Liposomes and Pm‐CM‐Liposomes were immobilized on poly‐L‐lysine coated glass within 3 days of preparation and imaged in PBS by AFM.^[^ ^60^ ^]^ Continuous images were made while the imaging force was increased by 10 pN between each image (**Figure** **3**a).^[^ ^21^ ^]^ The heights of each particle at increasing imaging force were tracked (Figure S5, Supporting Information, see Experimental Section for details). Consistent with the DLS results (Figure S1, Supporting Information), Pm‐CM‐Liposomes made with MS5 plasma membrane appeared smaller than Pm‐Liposomes (Figure 3b,c). The similarity in relative deformation under the influence of increased force indicated comparable mechanical properties for Pm‐Liposomes and Pm‐CM‐Liposomes (Figure 3d). Overall these results suggested that, contrary to what was observed previously when using K562 leukemia cell membrane to make Pm‐CM‐Liposomes (Figure S6, Supporting Information),^[^ ^9^ ^]^ inclusion of 10% bone marrow stromal cell (MS5) components into liposomes did not alter the mechanical properties of the resulting bilayer (Figure 3), nor favor interactions with the leukemia cells (Figure 2; Figures S2–S4, Supporting Information).

### 2.3 Preparation and Characterization of Rigid Cell Membrane Coated Nanoparticles

As a next step, based on these results, we prepared rigid cell membrane nanoparticles in order to test whether a higher nanoparticle rigidity favored the interaction between cell membrane nanoparticles and the targeted cells. To this end, silica cores were selected as a model core on which to deposit the bilayer in order to increase the rigidity of all compositions.

**Figure 4:**
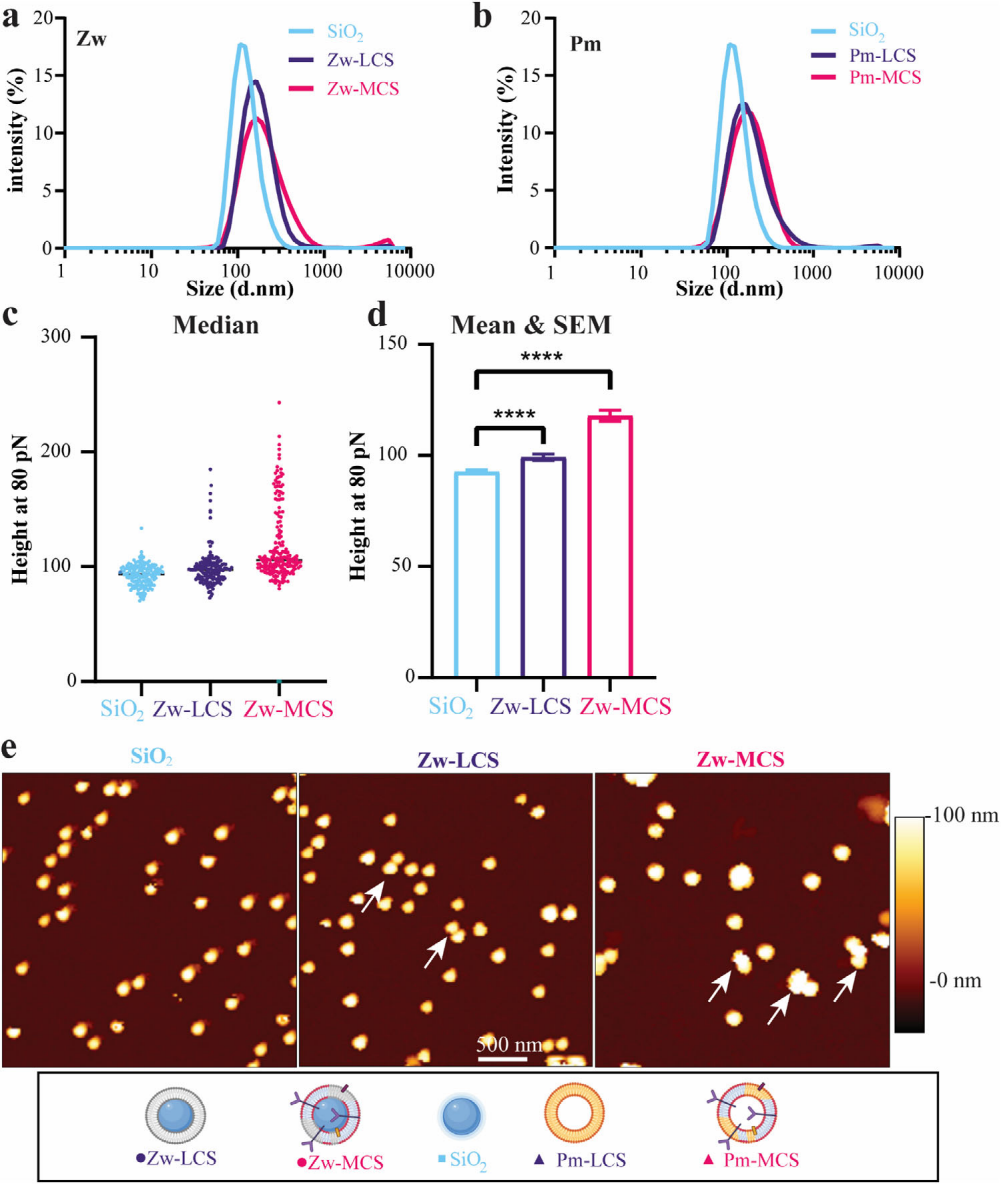
Characterization of Liposome‐ and CM‐Liposome‐coated silica (LCS and MCS, respectively). a,b) Average size distribution by DLS of (a) all coated Zw batches (*n* = 6, each measured in triplicate), and (b) all coated Pm batches (*n* = 4, triplicate) in low ionic strength water (25 µg mL^−1^) with SiO_2_ as reference (*n* = 5, triplicate). c) Median and individual heights recorded by AFM at 80 pN for bare silica cores, Zw‐LCS, and Zw‐MCS. d) Mean of heights and calculated SEM for the same samples (SiO_2_: *n* = 160; Zw‐LCS: *n* = 157, and Zw‐MCS: *n* = 190). A Welch and Brown‐Forsythe corrected ANOVA was used to compare the samples (adjusted *p* < 0.0001). e) Representative images of SiO_2_, Zw‐LCS, Zw‐MCS by AFM. Arrows indicate some nanoparticle clusters observed by AFM (see Figure S8, Supporting Information for further images and cluster quantification). Figure legends created with Biorender.com.

A ratio between 1:6 to 1:15 between the membrane protein and synthetic lipid amounts was suggested as optimal for CT26 membrane coating on mesoporous silica cores.^[^ ^25^ ^]^ In line with these indications as well as our previous work where K562 cell membrane was included in liposomes,^[^ ^9^ ^]^ we used a ratio of 1:10. Lipid‐coated silica (LCS) particles were prepared following published procedures.^[^ ^21^ ^]^ To prepare membrane‐coated silica (MCS), instead, CMLs and silica cores of ≈100 nm were sonicated and co‐extruded through a 200 nm filter. In both cases excess liposomes and CM‐Liposomes were removed by centrifugation.^[^ ^45^ ^]^ DLS, AFM, and Cryo‐EM were employed to characterize the different systems obtained. An overview of the measured characteristics of all the silica‐coated particles is given in **Table** **2**. As expected, bare silica particles were smaller than coated silica in both AFM and DLS measurements (**Figure** **4**; Figure S7, Supporting Information and summary in Table 2). Z‐average and size distribution were otherwise similar for all coated silica samples, with comparable PDI, which was slightly larger for Zw‐MCS (Figure S7, Supporting Information; Table 2). Upon deposition on a silica core to prepare LCS and MCS, zeta potential changes consistent with the deposition of the different types of bilayers were observed (Figure S7c, Supporting Information; Table 2). AFM and Cryo‐EM imaging were performed to verify and estimate coating efficiency.

**Table 2:** Zeta‐potential, size (Z‐average diameter), and polydispersity index (PDI) obtained by cumulant analysis of DLS data in low ionic strength water, together with AFM diameter and coating efficiency of silica, liposome coated silica (LCS) and cell membrane liposome coated silica (MCS) (Mean ± SD, Zw = 6 batcsilicahes, Pm = 4 batches, SiO_2_ = 5 batches, all measured in triplicate).

Particle	Zeta [mV]	Size [Z‐average]	PDI	Mean Diameter AFM [80pN]	Full %	Partial %
SiO_2_	−40 ± 7	117 ± 15	0.08 ± 0.05	92.6		
Zw‐LCS	−13 ± 4	162 ± 17	0.15 ± 0.04	99.2	11	7
Zw‐MCS	−25 ± 5	181 ± 35	0.23 ± 0.09	117.8	5	19
Pm‐LCS	−62 ± 2	167 ± 33	0.17 ± 0.06		20	13
Pm‐MCS	−41 ± 6	172 ± 13	0.19 ± 0.02		7	16

In relation to nanoparticle mechanical properties, compared with liposomes, silica has infinite rigidity, and membrane or lipid coating does not influence the rigidity of the resulting particles.^[^ ^24^ ^]^ Because of this, images at increasing forces of coated silica were not acquired.^[^ ^21^, ^24^ ^]^ Instead, for these samples AFM was used to image the particle population and measure the height of individual particles in order to gain insights on the deposition of bilayers. The power of AFM, here, is that it measures the actual height, and in the case of a non‐deformable sphere, such as the silica cores, AFM can accurately measure the diameter (or increase in diameter) per particle, as opposed to DLS, which gives an average of the hydrodynamic diameter of the total population with much lower resolution. The results showed that the Zw‐MCS size distribution included some larger sizes, suggesting the presence of some bigger objects (Figure 4c). Indeed, small clusters were observed in the AFM images (see arrows to indicate some examples in Figure 4e, as well as images and quantification for both Zw‐LCS and Zw‐MCS in Figure S8, Supporting Information). Overall, the whole distribution of both LCS and MCS shifted to larger sizes compared to silica, suggesting successful membrane coating on most particles (Figure 4c). In fact, Zw‐LCS were 3.9 nm bigger than bare silica (97.3 and 93.4 nm median height, respectively) which is consistent with the deposition of a bilayer. Instead, the median height of Zw‐MCS was 105.9 nm, which is 12.5 nm bigger than bare silica (Figure 4d). The slightly larger shift in size in respect to what was observed upon deposition of a simple bilayer is consistent with the deposition of a bilayer including membrane proteins (Figure 4c,d).^[^ ^18^ ^]^

### 2.4 Coating Efficiency and the Influence of Membrane Fluidity

**Figure 5:**
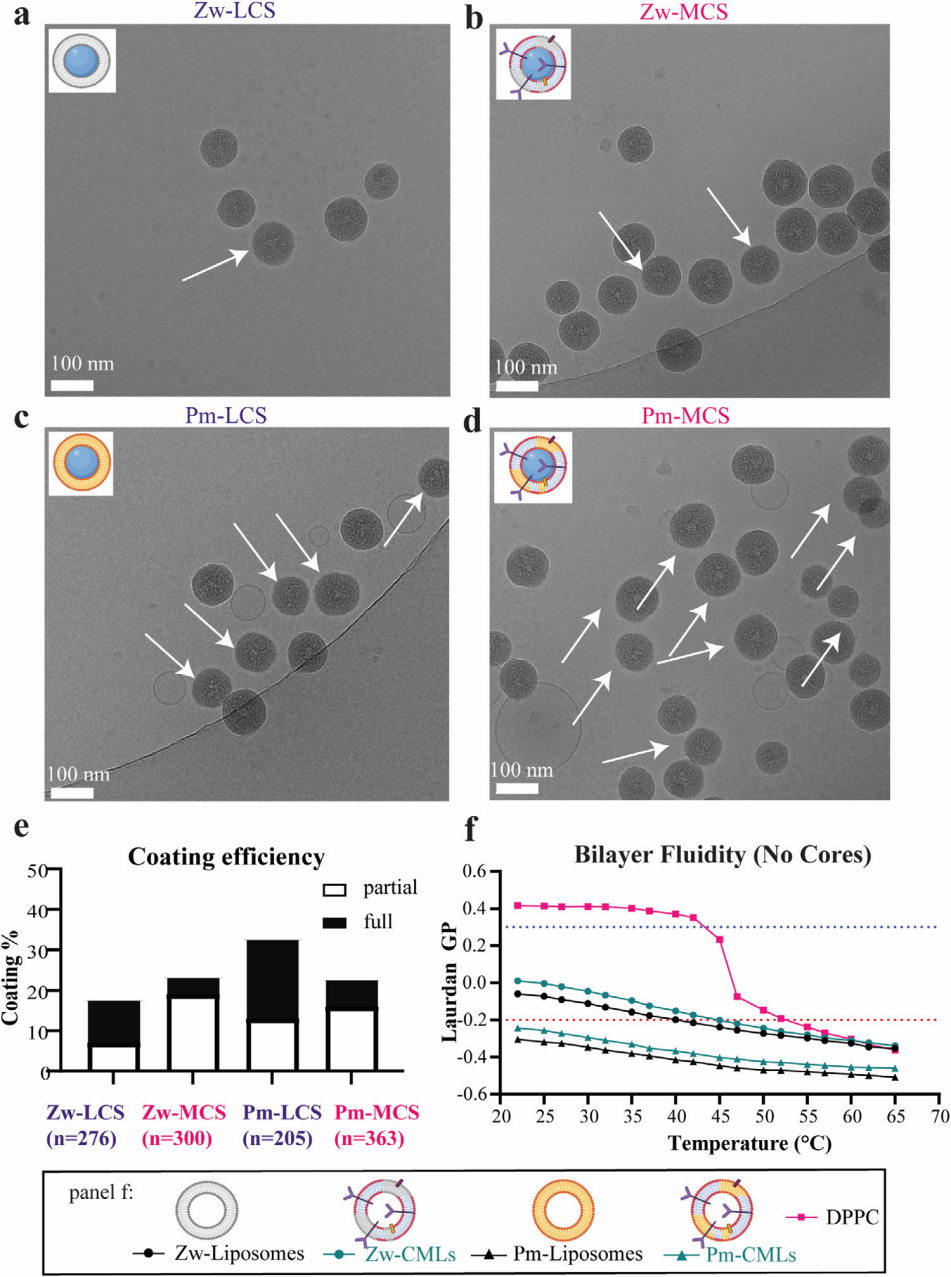
Coating efficiency by cryo‐EM imaging and membrane fluidity of LCS and MCS. Representative Cryo‐EM images of a) Zw‐LCS, b) Zw‐MCS, c) Pm‐LCS and d) Pm‐MCS. Areas where coating is visible are highlighted by white arrows. e) Estimation of coating efficiency for LCS and MCS particles from Cryo‐EM images of a representative batch of particles (Zw‐LCS: *n* = 276, Zw‐MCS: *n* = 300, Pm‐LCS: *n* = 205, Pm‐MCS: *n* = 363). f) Laurdan GP ratio obtained at increasing temperature for the different formulations (Mean, *n* = 2, from 1 batch of particles). Pure DPPC was included as a control (DPPC). A lower GP indicates higher bilayer fluidity. Figure legends and inser ts created with Biorender.com.

To further characterize the liposome and membrane‐coated silica, cryo‐EM was used to image the nanoparticles and confirm lipid bilayer deposition, while a Laurdan assay was used to compare the bilayer fluidity for the different formulations.^[^ ^19^ ^]^ Cryo‐EM images of the LCS and MCS particles showed that the samples included partially coated cores and fully coated cores (**Figure** **5**a–e). The amount of empty liposomes was relatively small (by AFM and Cryo‐EM), consistent with the washing performed to reduce their presence. The different formulations were stable in the presence of 10% FBS (Figure S9, Supporting Information). The particles included in several images were manually classified as partially or fully coated to estimate their fractions in a simplified manner (Figure 5e). However, we stress that the number of the partially coated particles is likely to be underestimated, since an apparently uncoated particle may have partial coating on a different plane.

Bilayer fluidity has been shown to aid in coating nanoparticles, allowing the membrane to more easily wrap around the core and overcome the energy needed to fuse patches with each other.^[^ ^25^ ^]^ Laurdan is a fluorescent probe used to distinguish differences in phospholipid order due to changes in membrane fluidity (see Experimental Section for details).^[^ ^61^, ^62^ ^]^ Here, measurements of Laurdan fluorescence were used to compare the bilayer fluidity of the starting liposomes and CMLs prior to deposition on silica (Figure 5f).^[^ ^62^ ^]^The results indicated that the zwitterionic bilayer (Zw) was less fluid than the plasma membrane‐like bilayer (Pm). Furthermore, the fluidity of the CM‐Liposomes after inclusion of membrane components was lower than for liposomes. A lower fluidity was expected upon inclusion of saturated lipids and cell membrane proteins into the bilayer of CM‐Liposomes. Therefore, the Laurdan results were consistent with the inclusion of membrane components in the bilayer. Interestingly, for the Pm‐like compositions that had higher fluidity (Laurdan GP < 0,2) in respect to the Zw compositions, a higher number of coated particles (partial and fully coated) was observed by cryo‐EM, as well (Figure 5e). Thus, cryo‐EM imaging and Laurdan measurements suggested that a higher bilayer fluidity facilitated deposition on a rigid core.

Though very difficult to quantify, the results also suggested that full coating of CM‐Liposomes on silica cores was observed only for a small fraction of particles: this is consistent with other studies attempting to deposit cell membranes on inorganic cores, which also reported that usually only partial coating can be obtained when the excess empty liposome and CM‐Liposomes are removed.^[^ ^45^, ^63^, ^64^ ^]^

### 2.5 Uptake of Rigid Cell Membrane Coated Nanoparticles in Leukemia Cells

**Figure 6:**
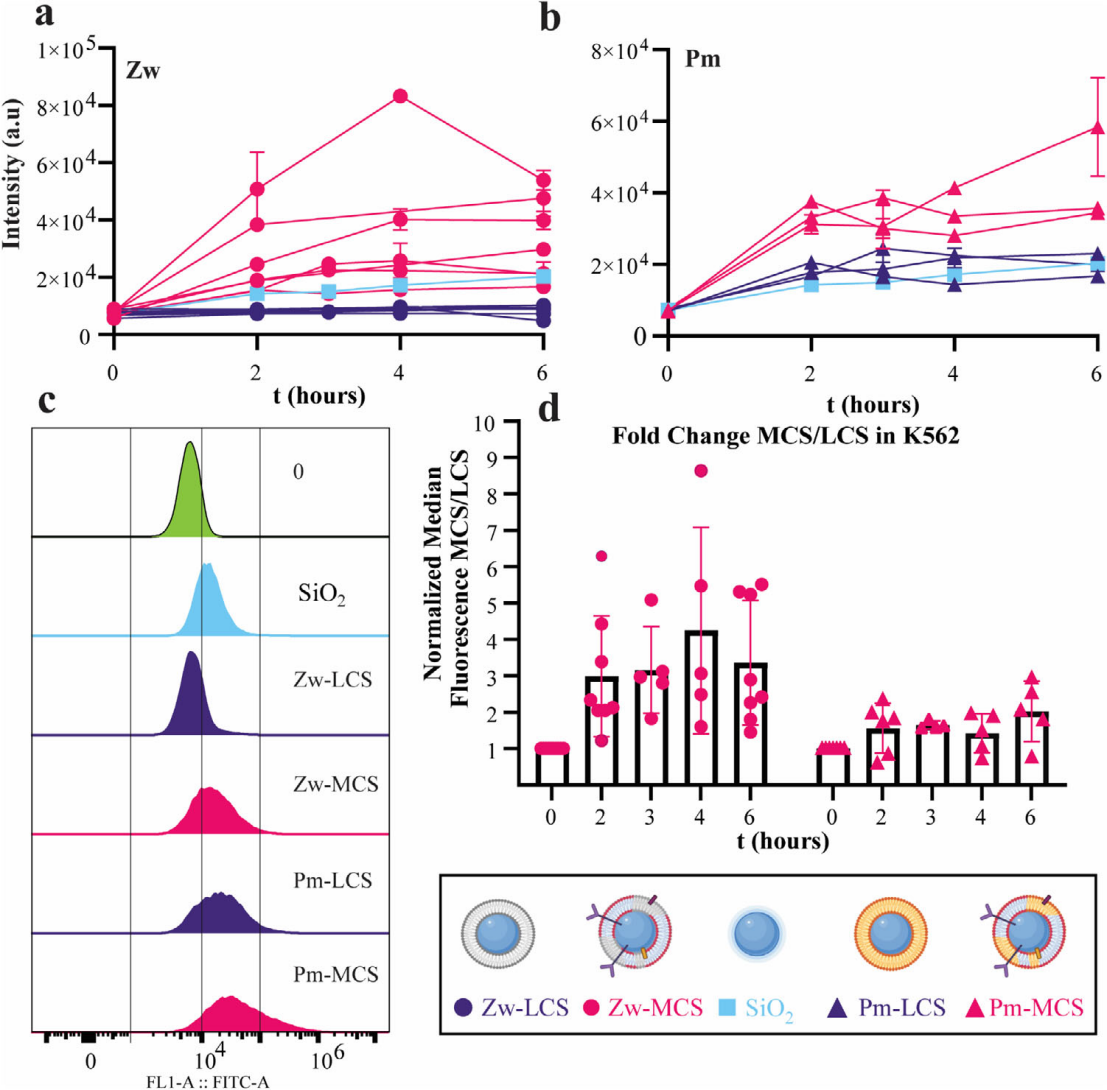
Uptake of LCS and MCS in K562 cells. a,b) Median fluorescence intensity and SD of K562 cells incubated with 50 µg mL^−1^ (based on silica concentration) of (a) Zw‐ and (b) Pm‐ LCS and MCS for increasing time. Uptake of bare silica was also included for comparison. Experiments were performed in duplicate and the average per experiment is shown (Mean ± SD, for a total of 6 and 3 independent kinetic experiments, in panel a and b, respectively). c) Staggered fluorescence distributions of K562 cells incubated with 50 µg mL^−1^ silica, MCS, and LCS in K562 for 2 hours. d) Ratio (Fold change) of the uptake of MCS in respect to LCS of same composition at various incubation times (from kinetic data shown in panels a‐b with the addition of other experiments with fewer timepoints). The normalized median cell fluorescence obtained in 10 (Zw) and 7 (Pm) experiments are shown together with their mean, and SD (Mean ± SD, *n* = 10 (Zw), 7 (Pm), each with duplicate samples). Figure legends created with Biorender.com.

As a next step, LCS and MCS were tested on leukemia cells to determine whether the deposition of cell membrane components on a rigid core affected the interaction with the targeted cells. K562 leukemia cells were incubated with the LCS and MCS with the different bilayer compositions, as well as with the bare silica as a reference for comparison (**Figure** **6**). In the case of the zwitterionic bilayers (Zw), Zw‐MCS were taken up more than bare silica and Zw‐LCS (Figure 6a). This was particularly surprising considering the strong negative zeta potential of bare silica nanoparticles in comparison to Zw‐MCS (Figure 4e). Also in the case of plasma membrane‐like bilayers (Pm), Pm‐MCS exhibited the highest accumulation in K562 in respect to Pm‐LCS and bare silica (Figure 6b), despite having the lowest (absolute) zeta potential (Figure 4e). When looking at the cell fluorescence distribution obtained by flow cytometry (Figure 6c), we noted that only for the samples with membrane components the width of the distribution increased and a tail on the right side of the distribution, thus a subpopulation of cells with higher uptake, was observed. Nanoparticle uptake by cells is known to be intrinsically heterogenous,^[^ ^65^, ^66^ ^]^ as indeed visible in all cell fluorescence distributions (Figure 6c). However, a broader distribution in comparison to what was observed for bare silica and LCS can be expected for the cell membrane nanoparticles, given the intrinsic heterogeneity in the membrane components included in individual nanoparticles within the sample.

Overall, the results showed that regardless of bilayer charge, both for Zw‐MCS and Pm‐MCS a higher uptake was observed than for Zw‐LCS and Pm‐LCS (silica coated with liposomes of same composition but without membrane components, Figure 6d). This behavior was confirmed for multiple batches of particles. These results indicated that even for partially coated silica, the presence of membrane proteins in the MCS enhanced the interaction with the cells,^[^ ^45^ ^]^ and led to higher uptake than what was observed for bare silica and LCS (despite these latter nanoparticles having a more negative zeta potential). This showed that nanoparticle rigidity strongly affects the interaction between cell membrane nanoparticle and the targeted cells, and only when using a rigid core, the inclusion of stromal cell membrane components favored uptake in the leukemic cells.

### 2.6 Interaction of Bone Marrow Stromal Cell Membrane coated Silica with Primary Leukemia Cells

As a final step, preliminary studies with primary cells from two leukemia patients were performed in order to gain first insights on whether the higher uptake observed for bone marrow stromal cell membrane‐coated silica in respect to liposome‐coated silica could be observed in more relevant cells as well. A first experiment was performed for nanoparticles added to the cells in 10% FBS (Figure S10a–d, Supporting Information, Patient 1). Cells were gated into live leukemia cells (blasts) using scattering, DAPI, and CD45 expression (Figure S10b, Supporting Information). Particles were simultaneously tested on K562 cells and primary cells, to confirm their activity in the model cell line (Figure S11, Supporting Information). For the primary cells, leukemic cells showed increased uptake of MCS over LCS for both lipid compositions (Figure S10c, Supporting Information), with stronger effects at higher concentration (Figure S10d, Supporting Information). These results confirmed what was observed previously in the K562 cell line model, and suggested that MS5 stromal cell coating might aid in reaching leukemic blasts, as long as the stromal proteins in the membrane nanoparticles are mechanically supported by a solid core.

Finally, in order to evaluate whether a higher uptake of MCS could also be achieved in leukemic stem cells, the cells that play a big role in the pathophysiology of leukemia,^[^ ^26^, ^27^, ^28^, ^29^, ^30^, ^31^, ^32^, ^33^, ^34^, ^35^, ^36^, ^37^ ^]^ primary cells from a second patient were exposed to bone marrow stromal cell MCS and LCS in 25% FBS medium (as usual for culturing them) (Figure S10e–i, Supporting Information, Patient 2). Cells were divided in lymphocytes and leukemia cancer cells (blasts) based on their scattering and CD45 expression. CD34 and CD38 staining were used to then subdivide the leukemia cells in leukemic stem cells (Q1‐Leukemia Stem Cells, CD34+, CD38 low) and leukemia progenitors (Q2‐Leukemia Progenitors, CD34+, CD38 high) (Figure S10f, Supporting Information).^[^ ^67^ ^]^ An increasing FBS concentration is known to usually decrease the interaction between nanoparticles and cells.^[^ ^68^ ^]^ Hence, as expected, the total uptake was lower at 25% FBS (Figure S10g, Supporting Information) than what was observed in 10% FBS (Figure S10a–d, Supporting Information, Patient 1). Of note, leukemic cells showed higher uptake than healthy lymphocytes, which might be explained due to lymphocytes’ poor capacity for nanoparticle uptake (Figure S10g, Supporting Information).^[^ ^69^ ^]^ After 4 hours, Zw‐MCS showed slightly higher accumulation in both leukemic stem cells and progenitors, which became larger over time (Figure S10h,i, Supporting Information). Overall, uptake of both particles in progenitor cells was higher than in the leukemia stem cells (Figure S10h,i, Supporting Information). These results, although preliminary, confirmed that also in primary cells from patients, inclusion of bone marrow membrane into the MCS promoted accumulation in leukemia cells.

While we realize that further studies in larger cohorts of primary AML patient samples are required to be able to generalize findings and obtain insights into nanoparticle adhesion properties in relation to specific genetic subtypes, our first aim here was to investigate whether the increased MCS particle uptake observed in leukemia cell lines could also be recapitulated in primary AML samples, which was the case. Similarly, the observed lower uptake in healthy lymphocytes in comparison to leukemic cells is consistent with the hypothesis that bone marrow MCS may be able to preferentially reach leukemia cells. However, further studies are required to demonstrate targeting in vivo as well as to ensure safety toward healthy cells.

## 3 Discussion

**Figure 7:**
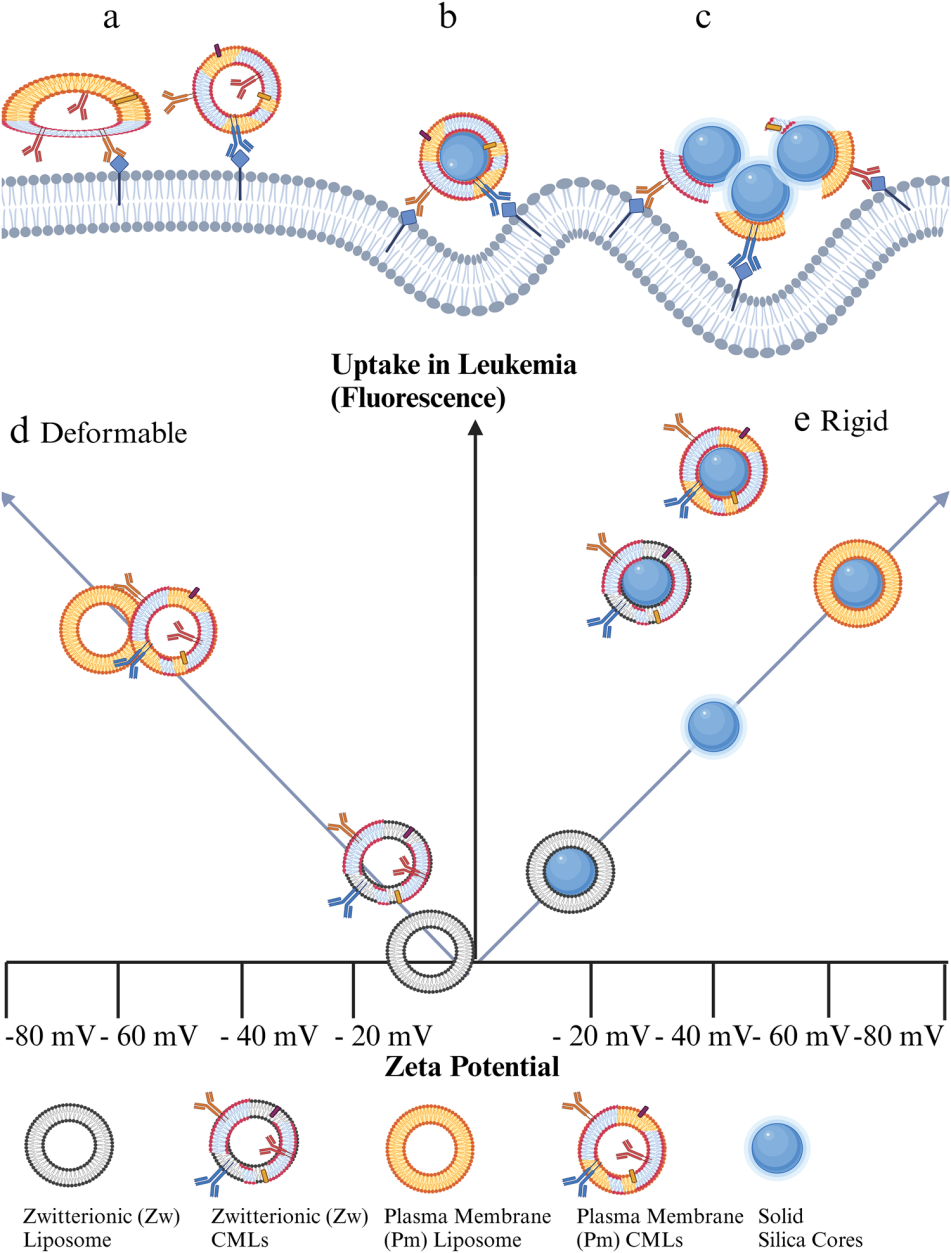
Schematic representation of the proposed mechanisms of interactions between soft and rigid cell membrane nanoparticles and targeted cells. a) A soft membrane particle has a lower capacity to bend the cell membrane for uptake. b) A rigid fully coated cell membrane particle may favor interaction with cell receptors and membrane bending for uptake. c) Agglomerates of partially coated cell membrane nanoparticles onto rigid cores may form and may promote membrane bending and interactions with multiple receptors on the targeted cells. d) Summary of the results reported in this work. Median cell fluorescence in K562 cells and zeta potential of the different particles studied (schematic). The blue line shows the expected trend in the results due to charge. Our results indicated that for deformable liposomes and CM‐Liposomes (CMLs, left side), uptake was higher for the more charged nanoparticles, but no difference was observed upon inclusion of cell membrane components. Instead for the more rigid LCS and MCS formed upon deposition of the bilayer onto silica cores, irrespective of particle charge, a higher uptake was observed upon inclusion of cell membrane components in the bilayer. These results suggested that a higher rigidity favors specific interactions between cell membrane nanoparticles and the targeted cells. Created in BioRender, de Weerd, S. (2024) https://BioRender.com/d93v796.

In a previous study, we found that incorporation of plasma membrane from K562 cells into bilayers led to the formation of CMLs with increased rigidity in comparison to liposomes without membrane components. The resulting K562 membrane CMLs showed higher uptake in both K562 cells and MS5 cells.^[^ ^9^ ^]^ Contrarily, in this work inclusion of stromal cell membrane led to the formation of soft CMLs of comparable rigidity than liposome of the same compositions but without membrane components (Figure 3). In this case, inclusion of membrane components did not enhance interaction with leukemia cells (Figure 2), possibly because the soft particles have a lower capacity to bend the cell membrane (**Figure** **7**a).^[^ ^22^ ^]^ Additional differences between the leukemia and stromal CMLs, for instance in protein and lipid composition, as well as murine versus human characteristics may play a role as well.

On the contrary, when depositing the bilayers onto rigid silica cores, the cell membrane nanoparticles showed higher uptake than the corresponding liposome‐coated silica without membrane components. Rigid nanoparticles may be able to bend the membrane and more easily wrap it around them, promoting uptake in the targeted cells (Figure 7b). Another possible interpretation is that the partially coated nanoparticles form small agglomerates, as indeed we observed by AFM (Figure 4h; Figure S8, Supporting Information). Small agglomerates have a bigger surface area, hence increased interactions with the targeted cells (Figure 7c). These two mechanisms, increased membrane wrapping and agglomerate formation, are possibly both at play, and together might explain why only with more rigid nanoparticles the inclusion of cell membrane components increased uptake in the leukemia cells in respect to the liposome and liposome‐coated silica. Further work is required to determine whether the same may be true for other types of cell membrane nanoparticles as well, and – more in general – to fully demonstrate whether presenting cell membrane components onto rigid nanoparticles is required to promote specific interactions with the targeted cells, as indeed our results suggest.

On another note, there is currently a debate on how best to deposit cell membrane components onto nanoparticles. The deposition of a cell membrane onto rigid cores is a dynamic process,^[^ ^70^, ^71^, ^72^ ^]^ and centrifugation to remove excess CM‐Liposomes generates shear forces that can strip the membrane coating from the cores. Some studies suggested that if the excess CM‐Liposomes are removed by washing, coating can be lost. Therefore, another way to prepare cell membrane‐coated nanoparticles is to leave an excess of CM‐Liposomes in the suspension, which would favor further bilayer deposition onto the nanoparticles.

Similarly, liposome size and the size of the cores are pivotal in determining the coating efficiency and may be further optimized, as also the choice of synthetic lipids, and the fluidity of the resulting bilayer.^[^ ^25^, ^42^ ^]^ Other studies suggested that the size of the vesicles and the cores should match to improve bilayer deposition,^[^ ^21^ ^]^ and buffer conditions can be optimized to enhance coating.^[^ ^21^ ^]^ Furthermore, it has been found that co‐extrusion of CM‐Liposomes and nanoparticle cores enhances deposition by generating shear forces around the nanoparticle.^[^ ^45^ ^]^

Another aspect that is debated in the field is whether small agglomerates of nanoparticles may be present, such as those observed in the Zw‐MCS images (Figure 4e; Figure S8a,b, Supporting Information): using EM, other researchers found that partially coated nanoparticles can form small clusters that are taken up in cells, irrespective of concentration.^[^ ^25^ ^]^ Formation and dissociation of small clusters is most likely a dynamic process, and AFM would capture such clusters once the particles get immobilized on the poly‐L‐lysine surface. Indeed, using AFM, we observed more dimers and trimers for the Zw‐LCS and in particular for Zw‐MCS particles (for which hexamers were also observed) compared to bare silica particles (Figure S8b, Supporting Information). Given the low density of the single particles and clusters on the AFM images, especially for the Zw‐MCS particles (Figure S8b, Supporting Information), it is likely that the clusters were already present in the solution before adhering to the surface. However, how the interaction of clusters with cells differs to single nanoparticle interactions needs to be studied. Similarly, additional work is necessary to gain more mechanistic insights on how cell membrane nanoparticles interact with the targeted cell and, for instance, to determine the contributions of specific receptors.

Additionally, in order to further develop stromal cell membrane nanoparticles for drug delivery applications, several other aspects need to be optimized. First, while here silica cores were used as a model to increase nanoparticle rigidity, a different core more suited for drug delivery applications could be selected. For instance, mesoporous silica is highly applied in drug delivery because their pores can be used to load high amounts of drugs.^[^ ^73^, ^74^ ^]^ Alternatively, by changing lipid composition, more rigid cell membrane liposomes could be obtained without the need of a nanoparticle core.^[^ ^75^ ^]^ Second, human stromal cell lines could be used to extract the cell membrane for nanoparticle preparation, and are likely to further improve interactions with human leukemia cells.

Next to this, the intracellular fate of the nanoparticles and release properties inside the targeted cells need to be determined, as also their in vivo distribution, liver accumulation, targeting capacity, and safety toward healthy cells. In fact, while we realize that also normal hematopoietic stem cells (HSCs) can interact with nanoparticles coated with stromal cell membranes, various labs have described that the AML cells have altered, and oftentimes stronger interactions with stromal cells, and while cobblestone areas (CAFCs, cobblestone area forming cell) induced by normal HSCs typically only form after 5 weeks on MS5, leukemic CAFCs form typically much earlier, within weeks, and have replating capacity, indicative of self‐renewal.^[^ ^35^, ^36^, ^49^, ^50^, ^56^ ^]^ Hence, we expect bone marrow stromal cell membrane nanoparticles to preferentially target the leukemic cells. Consistent with this, the preliminary tests on patient samples indicated that uptake of the bone marrow stromal MCS in the healthy lymphocytes was lower than in the leukemia cells (Figure S10g, Supporting Information). Nevertheless, for further development of stromal cell membrane nanoparticles for drug delivery, the interaction with healthy cells needs to be carefully monitored to ensure safety. Similarly, further studies are required to test their targeting capacity in vivo, as well as to determine overall whether stromal cell membrane or more classic approaches using leukemia cell membrane for homotypic targeting may be more suited.^[^ ^5^, ^7^, ^8^, ^9^ ^]^

## 4 Conclusion

In this work, we studied the effect of nanoparticle rigidity on the interactions between stromal cell membrane nanoparticles and leukemia cells.^[^ ^9^ ^]^ Inclusion of stromal cell membrane into liposomes led to the formation of soft CM‐Liposomes of comparable rigidity than liposomes of the same composition but without membrane components and did not enhance interaction with leukemia cells.^[^ ^22^ ^]^ However, upon deposition of the stromal cell membrane liposomes onto more rigid silica cores, higher uptake was observed in respect to the corresponding liposome‐coated silica without membrane components (Figure 7b,e). These findings clearly show that nanoparticle rigidity strongly affects the interactions between cell membrane nanoparticles and the targeted cells. Therefore, nanoparticle mechanical properties need to be carefully optimized in order to achieve cell membrane formulations with the required characteristics for targeted delivery.

## 5 Experimental Section

### Cell Culture

K562, MOLM13 and THP‐1 cells were obtained from DSMZ (Braunschweig, Germany) and maintained in RPMI 1640 medium containing 2 mm GlutaMAX (Gibco, ThermoFisher Scientific, UK), supplemented with 10% fetal bovine serum (FBS, Gibco, ThermoFisher, Brazil), 2 mm L‐glutamine, and 20 mm HEPES, at 37 °C in a 5% CO_2_ atmosphere. Murine stromal (MS5) cells, also acquired from DSMZ, were cultured in alpha MEM (Gibco, ThermoFisher Scientific, UK) with 10% FBS under the same conditions. All cell lines were maintained for fewer than 20 passages and were not used until after 4 passages. Mycoplasma testing was conducted regularly to ensure the absence of contamination.

### Plasma Membrane Purification

(Note: All centrifugations and buffers used were at 4 °C to prevent protein degradation.) At least 250 million MS5 cells were washed with PBS and then detached with 2 mm EDTA in PBS for 30 min before scraping or detached by scraping in PBS. Then, cells were collected in 50 mL Falcon tubes by centrifugation at 300 x g and washed once more with PBS. Afterward, cells were washed once in isotonic starting buffer (SB, 30 mm Tris‐Base, 225 mm mannitol, 75 mm sucrose in distilled water, adjusted to pH 7.4 with HCl, 4 °C). The washed cells were placed in 35 mL isotonic isolation buffer (IB, 30 mm Tris‐Base, 225 mm mannitol, 75 mm sucrose, 2.5 mm MgCl_2_, 0.5 mm EGTA, 1 cOmplete Roche EDTA free protease inhibitor cocktail in distilled water, adjusted to pH 7.4 with HCl, 4 °C) and deposited into a nitrogen cavitation vessel (Parr Instruments, USA). The vessel was pressurized with N_2_ to 200 Psi (or 280 Psi if EDTA was omitted for harvesting). After 30 min of equilibration, the vessel was depressurized once or twice until ≈80% lysis percentage was obtained and the homogenate was collected in a pre‐chilled 50 ml Falcon tube (the percentage of lysis was calculated by counting intact cells with a hemocytometer using phase‐contrast microscopy after cavitation;, one T175 was expected to have ≈8‐10 million MS5 cells). Then, unbroken cells and nuclei were removed by centrifugation at 800 × g for 5 min of the homogenate twice and the supernatant kept. The supernatant was then centrifuged twice at 10000 × g for 10 min in a SW31‐Ti (Beckman & Coulter) using pre‐cooled 38.5 mL Ultra Clear Ultra centrifuge tubes (Beckman Coulter, 38.5 mL Open‐Top Thinwall Ultra‐Clear Tube, 25 × 89 mm) to remove most of the mitochondria. To collect the plasma membrane and other membrane fragments, the supernatant was centrifugated at 41000 x g for 30 min in a fresh 38.5 mL ultracentrifuge tube. Another centrifugation at 41000 x g for 30 min followed by resuspension in isotonic buffer in another fresh 38.5 mL tube was employed to remove cytosolic impurities. The pellet was now dissolved in plasma membrane resuspension buffer (PMRB, 5 mm Bis‐Tris, and 0.2 mm EDTA in distilled water, adjusted to pH 6.0 by HCl, 4 °C) by a couple strokes of a Potter‐Elvehjem homogenizer with a tight‐fitting pestle, and layered on top of a 4 mL 38%, 3 mL 43% and 2.5 mL 53% wt/wt sucrose in PMRB discontinuous sucrose gradient made in a 13.2 mL, Beckman & Coulter, Open‐Top Thinwall Ultra‐Clear Tube, 14 × 89 mm and centrifuged at 95000 × g for 2.5 h in a SW41‐Ti (Beckman & Coulter). The band in the top of the 38% fraction corresponds to the band with purified plasma membrane (as tested by western blot analysis of plasma membrane markers). This fraction was collected by penetrating the bottom of the tube with a hot needle and collected in Eppendorf tubes. The fraction of interest was then placed in a fresh 13.2 mL tube and diluted with isotonic buffer SB and centrifuged at 95000 × g for 50 min to remove the sucrose originating from the gradient. The pellet was stored in storage buffer (30 mm Tris‐Base, 225 mm mannitol, 75 mm sucrose, and 1 cOmplete Roche EDTA free protease inhibitor cocktail in distilled water, adjusted to pH 7.4 with HCl, 4 °C), aliquoted in single or double use aliquots and snap frozen by lowering the closed Eppendorf tube in liquid nitrogen. The purified plasma membrane vesicles were stored at −80 °C for 6 months maximum and protein concentrations were determined using the modified DC Protein Assay (Bio‐Rad). Vesicles were defrosted before use.

### Protein Quantification

A modified version of the Bio‐Rad DC Protein Assay was utilized, incorporating SDS to solubilize membrane proteins due to the high lipid content in the samples, which can interfere with protein detection. Standard were prepared in the same buffer used for the samples, with bovine serum albumin (BSA, 98% purity, ThermoFisher Scientific, UK) at concentrations between 0.0625 and 2 mg mL^−1^. The assay was performed by following the manufacturer's protocol with the addition of 2% SDS to reagent B. Reagent S was also included to account for the presence of detergent. After 15 min at room temperature, absorbance was measured at 650 nm using a ThermoMAX microplate reader. Protein concentrations in unknown samples were subsequently determined using the corresponding calibration curves.

### SDS‐Page, Western Blot and Silver Staining

7.5 or 10% SDS‐PAGE gels were made and loaded with equal amounts of proteins in each lane according to the Bio‐Rad DC Protein Assay. The crude fraction was the pellet obtained from the cell lysate after removal of nuclei and mitochondria and differential centrifugation at 41000 × g, while the purified membrane was the fraction obtained after sucrose density fractionation of the crude pellet.^[^ ^4^, ^9^ ^]^ The proteins were denatured by mixing with 4x diluted loading buffer and heating at 95 °C for 10 min. A total of 35 µL of the diluted sample was loaded onto the gel and electrophoresis was run at 120 V. 10 µg of protein was loaded per lane. PVDF membranes were stained with ATP1a1 (ATPase Na+/K+‐transporting subunit alpha 1, as a marker for plasma membrane), TfR (Transferrin Receptor, plasma membrane), ATP5a1 (ATP synthase F1 subunit alpha, mitochondrial membrane), and GAPDH (glyceraldehyde‐3‐phosphate dehydrogenase, cytosol) in order to determine the composition of the different fractions and confirm isolation of a pure membrane fraction. For antibody detection, proteins were transferred to methanol‐activated PVDF membranes according to the manufacturer's instructions, for 1.5 h at 120 V in a cooled transfer tank. The membranes were then incubated in blocking buffer (20 mm Tris‐base, pH 7.6, 1.5 M NaCl, 0.1% Tween‐20, and 5% nonfat dry milk). Primary antibodies were added to the blocking buffer as follows: rabbit polyclonal anti‐ATP5a1 (1:2000, Novus Biologicals, Catalog number: NBP2‐92928‐0.1 mL, USA), mouse monoclonal anti‐transferrin receptor (1:2000, ThermoFisher Scientific, Catalog number: 13–6800), rabbit monoclonal anti‐GAPDH (1:2000, Cell Signaling Technology, Catalog number: 51745, Leiden, The Netherlands), and rabbit polyclonal anti‐ATP1A1 (1:2000, Proteintech, Catalog number: 55187‐1‐AP, USA). Secondary antibodies used included goat anti‐rabbit HRP‐conjugated secondary antibody (1:2000, Southern Biotech, Catalog number: 4049‐05, USA) and rabbit anti‐mouse HRP‐conjugated secondary antibody (1:2000, Southern Biotech, Catalog number: 6175‐05, USA). The signal was detected with an ECL detection kit (GE Lifesciences) according to the manufacturer's instructions, and blots were imaged with a ChemiDoc XRS (Biorad, USA). The original 16‐bit.tiff files were exported and then processed with ImageJ by converting to 8‐bit with linear scaling and inverting the images, which were then arranged into the figures using Adobe Illustrator 2022.

### Liposome and CML Preparation

The preparation of liposomes and CML was carried out using the thin‐layer evaporation and extrusion method. Lipids were obtained from Avanti Polar Lipids (Alabaster, Alabama, USA). The lipids used in this study include 1,2‐dioleoyl‐sn‐glycero‐3‐phosphocholine (DOPC 18:1 PC (cis)), 1,2‐dioleoyl‐sn‐glycero‐3‐phosphoethanolamine (DOPE, 18:1 PE), 1,2‐dioleoyl‐sn‐glycero‐3‐phospho‐(1′‐rac‐glycerol) sodium salt (DOPG, 18:1 (Δ9‐Cis) PG), cholesterol, and the fluorescent lipid 1,1′‐Dioctadecyl‐3,3,3′,3′‐Tetramethylindocarbocyanine Perchlorate (DiI, Sigma–Aldrich, The Netherlands). Lipids were dissolved in chloroform (Sigma–Aldrich, The Netherlands), aliquoted at a concentration of 10 mg mL^−1^, and stored at −20 °C. The appropriate amounts of lipids were added to glass vials, combined, dried under nitrogen, and left under vacuum overnight. The resulting lipid film was then rehydrated in filtered PBS (Whatman FP30/0.2, 0.2 µm filters) containing 0.05% Sodium Azide (Sigma–Aldrich, The Netherlands), with or without the addition of MS5 purified plasma membrane at a 1:10 protein‐to‐synthetic lipid ratio (wt/wt). The lipid film was gently shaken for 2–3 h at room temperature until fully dispersed. The sample was then freeze‐thawed 8 times using liquid nitrogen and room temperature water, followed by passage through a 0.2 µm polycarbonate filter (Avanti Polar Lipids) 21 times using the Avanti Mini‐Extruder (Avanti Polar Lipids). The samples were stored at 4 °C and used within 4 weeks.

### Low Ionic Strength Water

ROug1.3 mm H_2_PO_4_ was prepared in distilled filtered (Whatman FP30/0.2, 0.2 µm filters) water and brought to pH 7.4 with 1 m NaOH.

### Generation of LCS

Liposomes for LCS preparation were generated as descrived above, with the exception that the vesicles were sonicated in a bath sonicator for 10 min at room temperature instead of freeze thawed, and that DiI was omitted. After extrusion of the liposomes, plain 100 nm silica cores (plain, fluo‐green, 485/510 nm, 100 nm, 50 mg mL^−1^, 10 mL, suspension in water, from Micromod Partikeltechnologie GmbH, Germany, Catalog number:42‐00‐102) were added to 1 mg of liposomes in a 1:1 wt/wt lipid:silica ratio. The particle dispersion was incubated in an Eppendorf tube at room temperature for up to 3 hile occasionally resuspended by pipetting up and down to keep particles in solution. Now, LCS were washed twice with *low ionic strength water* by centrifugation at 15000 x g, 15 min, 15 °C. The supernatants containing unbound liposomes were removed (care was taken to leave a little layer of water over the pellet) and the pellets were resuspended by gently pipetting up and down in a small volume of ≈100 µL using a 100 µL tip. LCS were stored in low ionic strength water at 4 °C and used within 5 days.

### Generation of MCS

CM‐Liposomes were prepared as described above. Freeze‐thaw cycles were replaced by sonication in a bath sonicator for 10 min and DiI was not included. 100 nm plain silica cores were added to the CML dispersion in a 1:1 wt/wt artificial lipid:silica ratio and incubated for 10 min. After resuspension of the pellet, the system was co‐extruded 21 times through a 0.2 µm polycarbonate filter using the Avanti Mini Extruder. After preparation, MCS were washed, centrifuged, and stored in the same way as LCS and used within 5 days of preparation.

### Lipid and Silica Core Quantification After Extrusion and Washing

A fluorescence assay was conducted to determine the concentration of lipids remaining after extrusion, using the fluorescent lipid DiI to quantify lipid loss during the process. Calibration curves were generated for each lipid composition after freeze‐thaw but before extrusion, using DiI as the fluorescent marker. The extruded samples were diluted 1:4 with PBS before being added to a 96‐well black plate (Greiner). Calibration standards ranged from 0.5 to 0.0625 mg mL^−1^ (higher concentrations exhibited reduced fluorescence. Fluorescence measurements were taken at room temperature, with excitation at 485 nm, emission at 555 nm, and a cutoff at 550 nm, using a SpectraMax Gemini XPS microplate spectrofluorometer. The lipid concentration was then calculated using the calibration curve. A similar approach was employed to assess the loss of silica cores during extrusion and washing. Plain 100 nm silica cores were washed by centrifugation at 15000 x g and resuspension in low ionic strength water twice to remove eventual free dye. No relevant loss of silica was observed and the concentration was set to 1 mg mL^−1^ and serially diluted for the calibration curve. The concentration of prepared LCS and MCS was calculated using this calibration curve.

### Size Distribution and Zeta Potential Measurements

Using a Malvern Zetasizer Nano ZS, the hydrodynamic diameter and zeta potential of the CM‐Liposomes and liposomes were determined. Dynamic light scattering (DLS) was employed to assess size distribution. Samples were diluted in PBS, low ionic strength water, or complete medium (containing 10% FBS) to a concentration of ≈25 µg mL^−1^ lipid, or 50 µg mL^−1^ when using complete medium or 25 and 100 µg mL^−1^ when using silica, respectively. Measurements were conducted at 25 °C using disposable folded DTS1070 capillary cells (Malvern Instruments Ltd., Worcestershire, UK). Each measurement was repeated three times, and their average was presented in the figures. In the case of liposomes, the refractive index for phospholipids was used and in the case of coated and non‐coated silica the refractive index of silica was used.

### Atomic Force Microscopy (AFM)

AFM experiments were conducted in liquid using Quantitative Imaging (QI) mode on a JPK Nanowizard ultra‐speed AFM. To immobilize nanoparticles, poly‐L‐lysine‐coated glass was freshly prepared according to a published protocol.^[^ ^60^ ^]^ Briefly, coverslips were cleaned with ethanol and hydrochloric acid (97%/3% v/v), then incubated in a 0.01 mg mL^−1^ poly‐L‐lysine solution for one hour. The coated glass was rinsed with deionized water and dried at 37 °C overnight. Liposomes and CM‐Liposomes were imaged within 4 days after preparation, here stock nanoparticle solution was diluted with PBS, and 50 µL of the sample (≈0.015 mg mL^−1^) was applied to the poly‐L‐lysine‐coated glass. The chamber was then filled with 450 µL PBS. For Zw‐LCS, Zw‐MCS, and SiO_2_ particles, ≈0.06 mg mL^−1^ silica was dissolved in 100 µL PBS and loaded on the glass after which the chamber was filled up with 400 µL PBS. Measurements were performed using qp‐BioAC CB3 probes (Nanosensors) with spring constants typically ranging between 0.03 and 0.09 N m^−1^. Prior to imaging, cantilevers were calibrated using the contact‐based and thermal noise methods provided in the JPK Nanowizard control software (version 6). QI images were captured at increasing forces (in steps of 10 pN, starting at 80 pN and going up to 170 pN) while keeping other imaging parameters constant (128 × 128 pixels, 3 µm x 3 µm, 15 ms pixel time, 300 nm lift height). Images were processed using JPK Data Processing software (version 6.1).

### Processing AFM Images and Automated Height Plot Extraction

Cleaned up images were loaded into Gwyddion (32‐bit, version 2.58, released 2021‐02‐09). In some occasions, stripes were present on the edge of the image (indicating loss of tip contact) and in these cases, they were removed with the crop tool. Grain analysis was performed by marking the grains using a ≈19 nm threshold. Edge‐touching grains were removed. Then, x, y, and z coordinates for each grain were extracted and tab‐delimited output files were generated. The file was manually curated and if height data from one particle gave multiple data points, the highest height was kept. In the case of LCS, MCS, and SiO_2_ particles, the amount of particles that presented as double, triple, etcetera were counted and excluded from the height data. Data points that, upon inspection of the image, were clearly not a particles were also excluded (artifacts on the side of particles for example). For the analysis of images at increasing forces, first, a script was developed in Python 3.12 to correct for the cantilever drift in the repeated AFM images. 30 nm was set as allowable drift radius. Then, to correct potential image drift across sequential images, a reference nanoparticle which (i) was present in all images, and (ii) was spatially isolated, with no neighboring particles, was selected. The coordinates of the selected reference nanoparticle were determined in each subsequent image to quantify the relative positional drift from its initial location in the first image. The calculated drift vector was then applied as a corrective offset to all other particle coordinates in each image, realigning them to a consistent spatial reference based on the first image and effectively eliminating drift‐induced artifacts. To extract the height plots, a script was developed that checked the drift‐corrected tab‐delimited files of each set of imaging forces, and matched the x and y coordinates of the particles, and placed the x, y, and z values of these corresponding data points in a new tab‐delimited file, tracking in this way the height of the particles at increasing imaging force. If an x, y coordinate was too far away (100 nm cut off) from the previous x and y coordinate at the previous imaging force, a new trajectory was created. The trajectories generated following this script were also checked manually, in order to confirm that the new trajectories were correct. Data points clearly coming from particles that adhered anew on a spot where a particle previously resided (which could be confused as the same nanoparticle) were removed from the analysis. After, the obtained heights were plot using Graphpad Prism (version 8.4.3 (686)). Normalized heights were calculated over all particles by dividing the current height of each particle by the height at 80 pN.

### AFM Cluster Counting

Cleaned up images were opened in the JPK Data Processing software (version 6.1) and the amount of single particles and clusters were counted by hand (only clusters and particles that were visible as a whole were included) and were reported using GraphPad Prism.

### Uptake Kinetics in Leukemia Model Cell Lines

CML and liposome uptake by cells was measured using flow cytometry. K562 cells were seeded at 50.000 cells well^−1^, THP‐1 at 150000 cells well^−1^ in 24 wells plates (Sarstedt) in 250 µL growth medium. MOLM13 cells were seeded at 50000 cells well^−1^ in 150 µL growth medium in 48 well plates (Greiner). After 24 h, nanoparticles were added in such a concentration in 100 µL to arrive at the necessary concentration noted in the figure legends. The plate was mixed by gently shaking the plate back and forward. Nanoparticle uptake was assessed in different cell‐lines at various incubation times. At the selected times, cells were harvested, transferred in FACS tubes, and washed with 500 µL PBS (2 times 300 x g, 5 min, RT), and finally resuspended in 100 µL PBS. Flow cytometry data was acquired using a Cytoflex S flow cytometer (Beckman Coulter). MOLM13 cell fluorescence was acquired using a Quanteon 2 (Quanteon). For energy depletion experiments, cells were incubated with nanoparticles in standard conditions or (after pre‐incubation with 5 mg mL^−1^ NaN_3_ in complete medium for 30 min)  in the presence of 5 mg mL^−1^ NaN_3_ in complete medium. Flow cytometry data was processed and analyzed using FlowJo software (version 10.8.1). To distinguish the cell population, forward scattering (FSC) was plotted against side scattering (SSC), and cell debris were excluded by applying gates. Additionally, cell doublets were excluded by gating on FSC‐H versus FSC‐A plots. Fluorescence was measured for 20000 cells per sample, with three replicates or duplicates prepared for each experimental condition. The median fluorescence intensity for each condition was averaged across the replicates at each time point. These data were then graphed using GraphPad Prism (version 8.4.3), each dot being the average of a triplicate or duplicate.

### Characterization of Nanoparticle Uptake Mechanisms with Transport Inhibitors

K562 cells were exposed to inhibitors of endocytosis before and during incubation with nanoparticles. Prior to the experiments, 50000 cells were seeded per well in a 24‐well plate (Sarstedt). 24 h after seeding, the cells were exposed to various inhibitors at different concentrations for 30 min in serum‐free medium (for dynasore) or complete medium (for nocodazole, Cytochalasin D, EIPA, and chlorpromazine).

The following conditions were found to be optimal and were used in K562: sodium azide (Sigma Aldrich) 5 mg mL^−1^; 5‐(N‐ethyl‐N‐isopropyl) amiloride (EIPA) (Tocris) 100 µm; Chlorpromazine hydrochloride (Sigma Aldrich) 10 µg mL^−1^; Dynasore (Biovision) 50 µg mL^−1^ and Nocodazole (Biovision) 5 µm; Cytochalasin D (Focus biomolecules) 2.5 µg mL^−1^. After preincubation, 20 µg mL^−1^ Pm‐CM‐Liposomes and Pm‐Liposomes in RPMI with 10% FBS were added to cells in standard conditions or in the presence of the different inhibitors.

### Determination of Bilayer Fluidity with Laurdan GP

For Laurdan experiments, liposomes and CM‐Liposomes were prepared as described above but without DiI. A 100 mm laurdan stock (Tocris Bioscience) was prepared in DMF. After, liposomes, CM‐Liposomes, and pure dipalmitoylphosphatidylcholine (DPPC in chloroform, Avanti Polar Lipids) liposomes as control were diluted to 0.2 mg mL^−1^ lipid and 5 µm laurdan was added to each well in a 96 well black plate with transparent bottom (Greiner). Emission spectra were captured between λ = 400 and 600 nm, excitation λ = 340 nm with a step size of λ = 10 nm, and bandwidth of λ = 9 nm in a Synergy H1 (BioTek) plate reader. Fluorescence spectra were read out at different temperatures ranging from 22 to 65 °C, allowing a 3 min equilibration time between each temperature. To calculate the general polarization as a measure of the bilayer fluidity, the GP_320_ was calculated using the following Equation (1):^[^ ^61^ ^]^

(1) $$\mathrm{GP}320=\frac{\left(\mathrm{Fluorescence}\;\lambda \;440-\mathrm{Fluorescence}\;\lambda \;490\right)}{\left(\mathrm{Fluorescence}\;\lambda \;440+\mathrm{Fluorescence}\;\lambda \;490\right)}$$

### Cryo‐EM

Samples (3 µL) with coated silica nanoparticles (≈1 mg mL^−1^) were loaded on a carbon‐coated copper grid (Quantifoil 3.5/1, Quantifoil Micro Tools) and rapidly frozen with liquid ethane using a Vitrobot (FEI). Images were acquired with a slow‐scan CCD camera under low‐dose conditions on a Tecnai T20 (FEI) cryo‐electron microscope operating at 200 keV, equipped with a Gatan model 626 cryo‐stage. Afterward, images were opened, and the particles were subsequently binned in the three situations: Full coating, partial coating, and no coating provided the particles were in full view, clearly visible, and not overlapping. The number of images analyzed were as follows: Zw‐LCS (38), Zw‐MCS (34), Pm‐LCS (33), and Pm‐MCS (33).

### Primary Cell Experiments

Bone marrow or peripheral blood samples of AML patients were studied after informed consent and protocol approval by the Ethical Committee of the UMCG in accordance with the Declaration of Helsinki (Approval number NL43844.042.13). Patient samples were thawed before the experiment and allowed to recuperate in T75 cells in alpha MEM (Gibco, ThermoFisher Scientific, UK) with 25% FBS in the presence of cytokines (G‐CSF, IL3, Romiplostin, SR1, and UM171) at 37 °C in a 5% CO_2_ atmosphere. After ≈48 h of recovery, cells were collected and resuspended in various concentrations of pre‐dispersed LCS, MCS, and bare SiO_2_ particles, and the cells were plated in 96 well plates (Greiner) at 125.000 cells well^−1^ in 250 µL alpha MEM supplemented with 10% or 25% FBS (and cytokines) with only one or two replicate samples, depending on the experiment and amount of cells recovered after defrosting. LCS and MCS were prepared the day before cell exposure. Particles were also exposed to K562 cells to control the efficacy of the particles used with the primary cells. At various time points, primary cells were harvested, placed in FACS tubes and pelleted by centrifugation at 450 x g for 5 min. Cells were washed with 2 mL of PBS with 0.5 mM EDTA, centrifuged, and stained in ice‐cold antibody staining mix comprising of PE‐Cyanine7 anti‐human CD45 (Biolegend catalog number: 304016), PE anti‐human CD34 (Biolegend catalog number: 343510), APC anti‐human CD38 (Biolegend catalog number: 303510), DAPI and Fc‐block in PBS at 4 °C for 30 min. For Patient 1, a small population of cells was identified that did not belong to the blast population based on scattering. Higher uptake from MCS over LCS was observed both for the main populations of cells and an undefined population detected in the forward‐side scattering plot (Figure S8c, Supporting Information). This undefined population of cells was excluded from the blast population as shown in the main figure. Cells were again washed with 2 mL of PBS with 0.5 mm EDTA, centrifuged at 450 x g for 5 min, resuspended in 100 µL PBS, and measured at a MACSQuant X flow cytometer (Miltenyi Biotec, Germany).

### Statistical Analysis

In Figure 2a, western blots were repeated to confirm the outcomes and showed similar results.^[^ ^9^ ^]^ Figure 2c shows the average and SD of the median cell fluorescence over three replicate samples (mean ± SD, *n* = 3) in 3 independent uptake kinetics experiments. Figure 2d shows the same for three independent uptake kinetic experiments, each with duplicate samples (mean ± SD, *n* = 2). In Figure 3, one example of an experiment is shown but it was repeated several times with similar results. In Figure 3b, extracted individual height values at 80 pN are shown together with their median (Pm‐liposome: median, *n* = 179 – Pm‐CML: median: *n* = 315), and in Figure 3c the mean and SEM of the same data were calculated and are shown (Pm‐liposome: mean ± SEM, *n* = 179 – Pm‐CML: mean ± SEM, *n* = 315). In Figure 3d, the extracted heights of each included particle at each imaging force was divided by the height at 80 pN to give the average normalized height per particle. This normalized height was then averaged over all the surviving particles per imaging force, and the SD was calculated (mean ± SD). In Figure 4a the average size distribution by DLS of 4 batches of Zw particles is shown (mean, *n* = 4), and in Figure 4b of 10 batches (mean, *n* = 4). The size distribution of SiO_2_ (mean, *n* = 5) is also shown as reference in a and b. In Figure 4c, the height of each individual particle in the AFM images is reported together with their median (SiO_2_: median, *n* = 160), (Zw‐LCS: median, *n* = 157), (Zw‐MCS: median, *n* = 190) whilst the mean and SEM of the same results are shown in Figure 4d (SiO_2_: mean ± SEM, *n* = 160), (Zw‐LCS: mean ± SEM, *n* = 157), (Zw‐MCS: mean ± SEM, *n* = 190). Due to the varying SD of the three samples, a Welch and Brown‐Forsythe corrected ANOVA was used for comparison (adjusted *p* < 0.0001). In Figure 5a–d a representative cryo‐EM image is shown of a total of: Zw‐LCS (38), Zw‐MCS (34), Pm‐LCS (33), Pm‐MCS (33) images. In these images *n* individual particles were analyzed (Zw‐LCS: *n* = 276, Zw‐MCS: *n* = 300, Pm‐LCS: *n* = 205, Pm‐MCS: *n* = 363), and subdivided as partial and full coating. Their fraction (%) in respect to the total population was calculated and is shown in Figure 5e. In Figure 5f Laurdan excitation was measured in duplicate samples and the GP_320_ value was calculated and is shown (mean, *n* = 2). Figures 6a,b show the average and SD over 2 replicate samples of the median cell fluorescence intensity (mean ± SD, *n* = 2) in 6 (Figure 6a) or 3 (Figure 6b) uptake kinetics experiments. The same results are shown in Figure 6d where the averaged fluorescence intensity obtained for Zw‐ and Pm‐MCS has been normalized for the averaged fluorescence intensity of the same formulation without membrane components (Zw‐ and Pm‐LCS, respectively), together with the average and SD of the normalized data obtained in all experiments. (mean ± SD, *n* depends on timepoint).

## Conflict of Interest

The authors declare no conflict of interest.

## Supporting information



Supporting Information

## Data Availability

The data that support the findings of this study are available from the corresponding author upon reasonable request.
