# Supplementary material for: Effect of Nanoparticle Rigidity on the Interaction of Stromal Membrane Particles with Leukemia Cells
Source: Adv Healthc Mater. 2025 Jun 8;14(19):2500667. doi: 10.1002/adhm.202500667 (PMC12304856; doi:10.1002/adhm.202500667)
Supplement: Supplementary file 1 — Supporting Information [file ADHM-14-0-s001.docx]

Supporting Information

Effect of Nanoparticle Rigidity on the Interaction of Stromal Membrane Particles with Leukemia Cells

Sander de Weerd, Xinyu Ma, Zahra Zohali, Lieve L. Oudejans, Emmanouil Kyrloglou, Marc C.A. Stuart, Wouter H. Roos*, Jan Jacob Schuringa*, Anna Salvati*


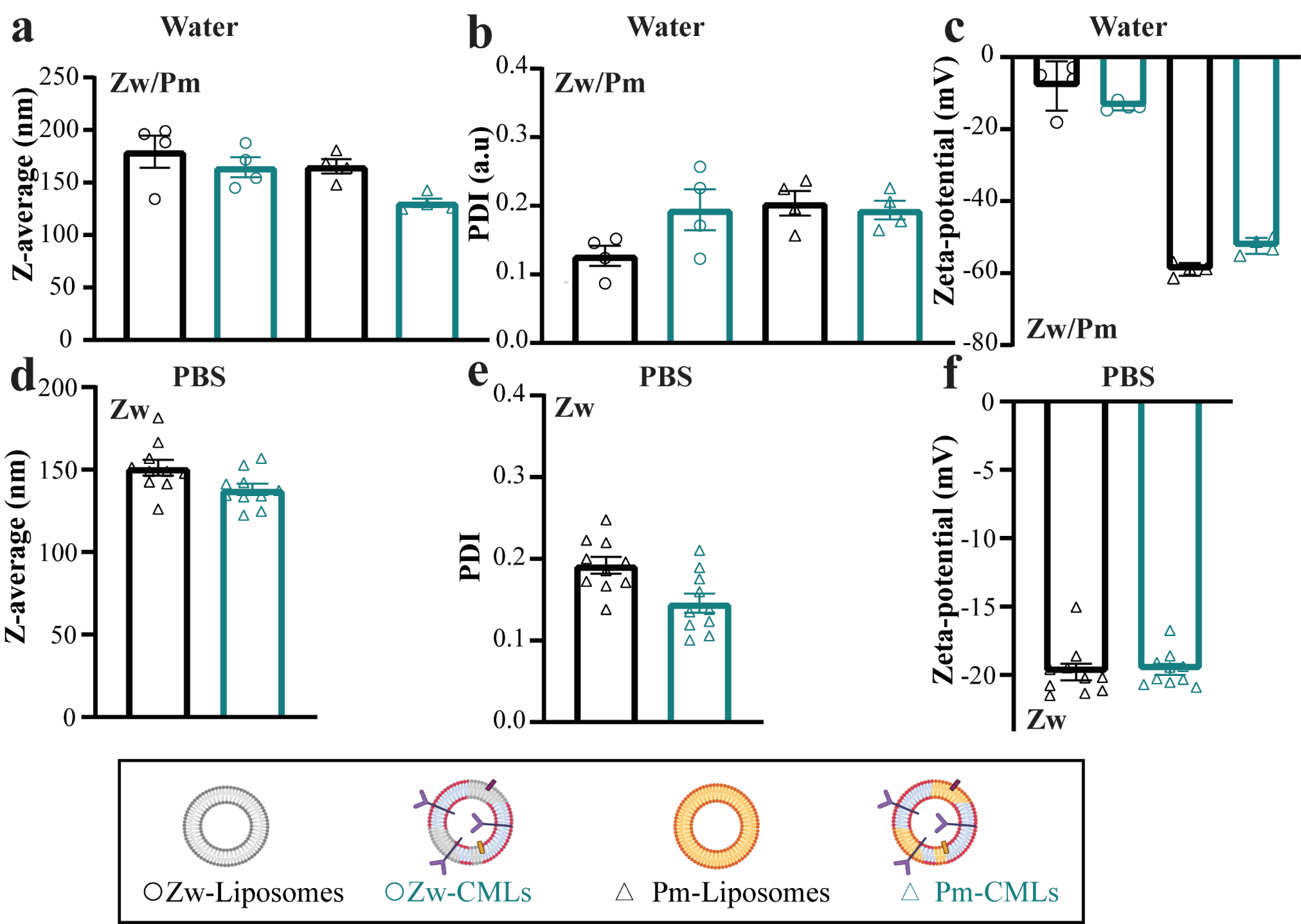


**Figure S1** - a) Z-average and b) PDI obtained by DLS and c) zeta potential of Zw-Liposomes, Zw-CM-Liposomes, Pm-Liposomes and Pm-CM-Liposomes measured in low ionic strength water. d) Z-average and e) PDI obtained by DLS and f) zeta potential of Pm-Liposomes and Pm-CM-Liposomes measured in PBS (25 µg/mL lipids). In panel a-f, each dot represents the results obtained for a different batch of particles, together with the average and SEM of all batches (mean ± SEM, n=4 and n=10 for a-c and d-f, respectively).


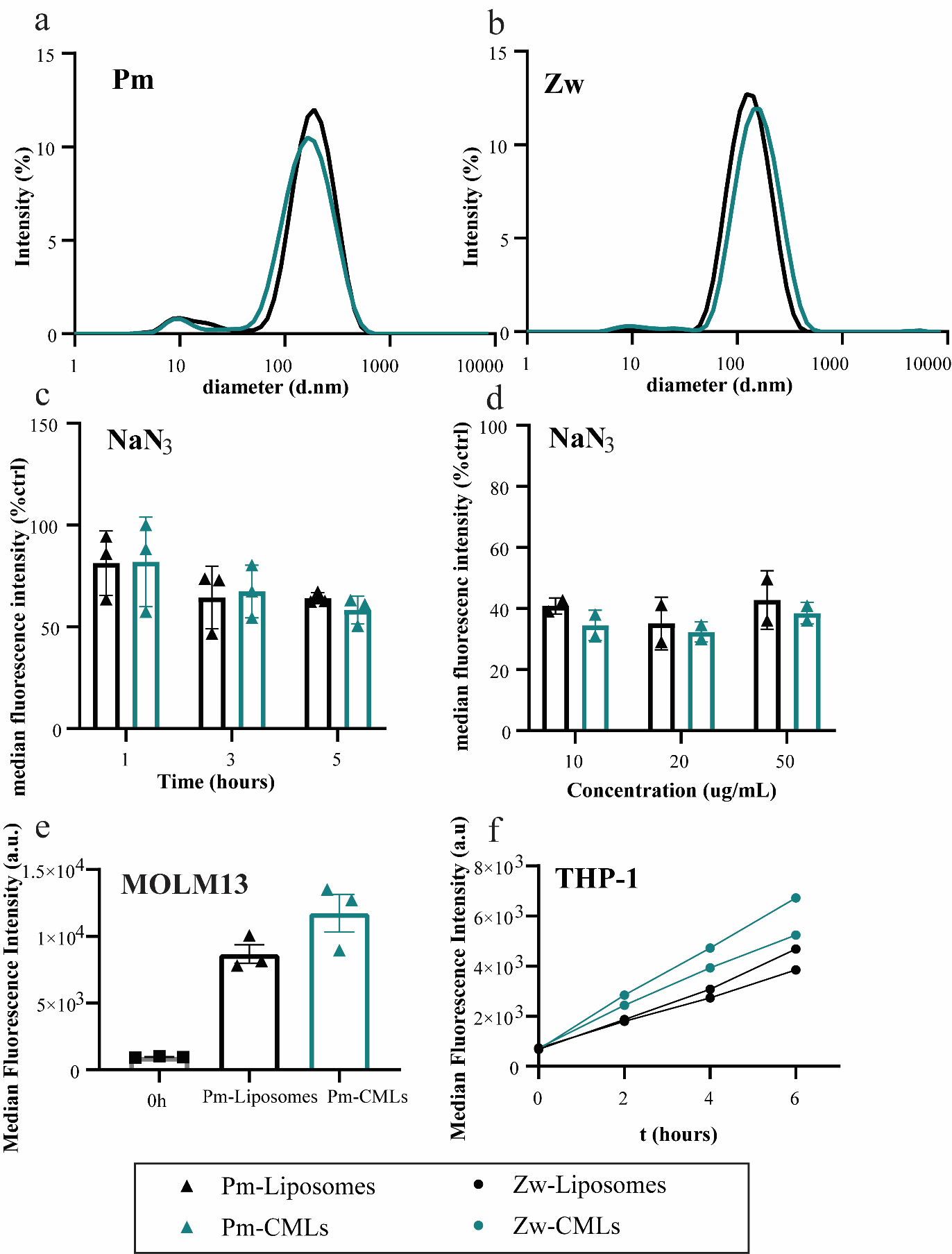


**Figure S2** – **Characterization and uptake behaviour of Pm and Zw liposomes and CM-Liposomes.** a-b) Size distribution by DLS of (a) Pm-compositions and (b) Zm-compositions in medium with 10% FBS (100 µg/mL, *n* = 3 and *n* = 1 for Pm and Zw respectively). c-d) Effect of energy depletion on nanoparticle uptake. K562 cells were incubated with Pm-liposomes and Pm-CM-Liposomes in the presence of 5 mg/mL sodium azide in 10% FBS (c) for increasing time at 10 µg/mL or (d) for 4 hours at increasing concentrations. Each data point is the average over 3 replicate samples in an independent experiment. The results are normalized by the uptake in cells incubated with the particles in standard conditions (% ctrl) (mean ± SD, n=3 independent experiments in c and n=2 in d). e) Uptake of Pm-Liposomes and Pm-CM-Liposomes in MOLM13 cells (50 µg/mL) in the presence of 10% FBS. Each data point is the average over 3 replicate samples in an independent experiment. One of the 3 experiments was performed with a single sample. The bar plot and error bars show the mean ± SEM of the results obtained in 3 independent experiments (n=3). f) Uptake of Zw-Liposomes and Zw-CM-Liposomes by THP-1 cells in 10% FBS at 50 µg/mL. Each data point is the average of duplicate samples (mean ± SD error bars are too small to see, n=2). The results of 2 independent experiments are shown.


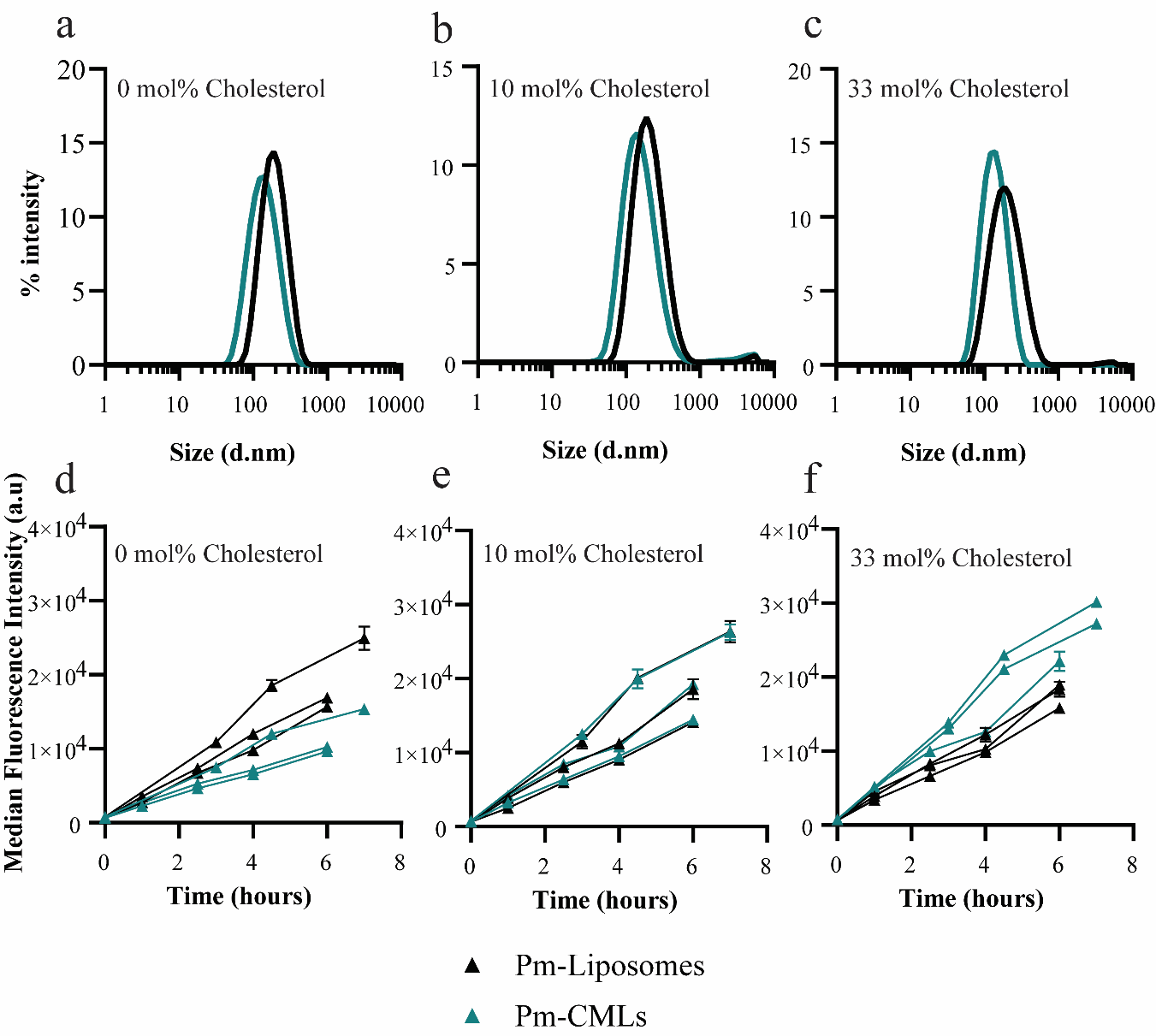


**Figure S3 – Size distribution and uptake kinetics of Pm-CM-Liposomes and Pm-liposomes with different cholesterol content. a-c)** Size distribution by DLS of a) 0 mol%, b) 10 mol%, c) and 33 mol% cholesterol Pm-compositions in PBS (25 µg/mL, n=1). d-f) Uptake in K562 cells incubated with 20 µg/mL Pm-Liposome and Pm-CM-Liposomes having d) 0 mol%, e) 10 mol% and f) 33 mol% cholesterol. Symbols represent the average of triplicate samples in 3 independent experiments (mean ± SD, n=3). Only for the 33 mol% cholesterol CM-Liposomes a slightly higher uptake was observed in comparison to liposomes.


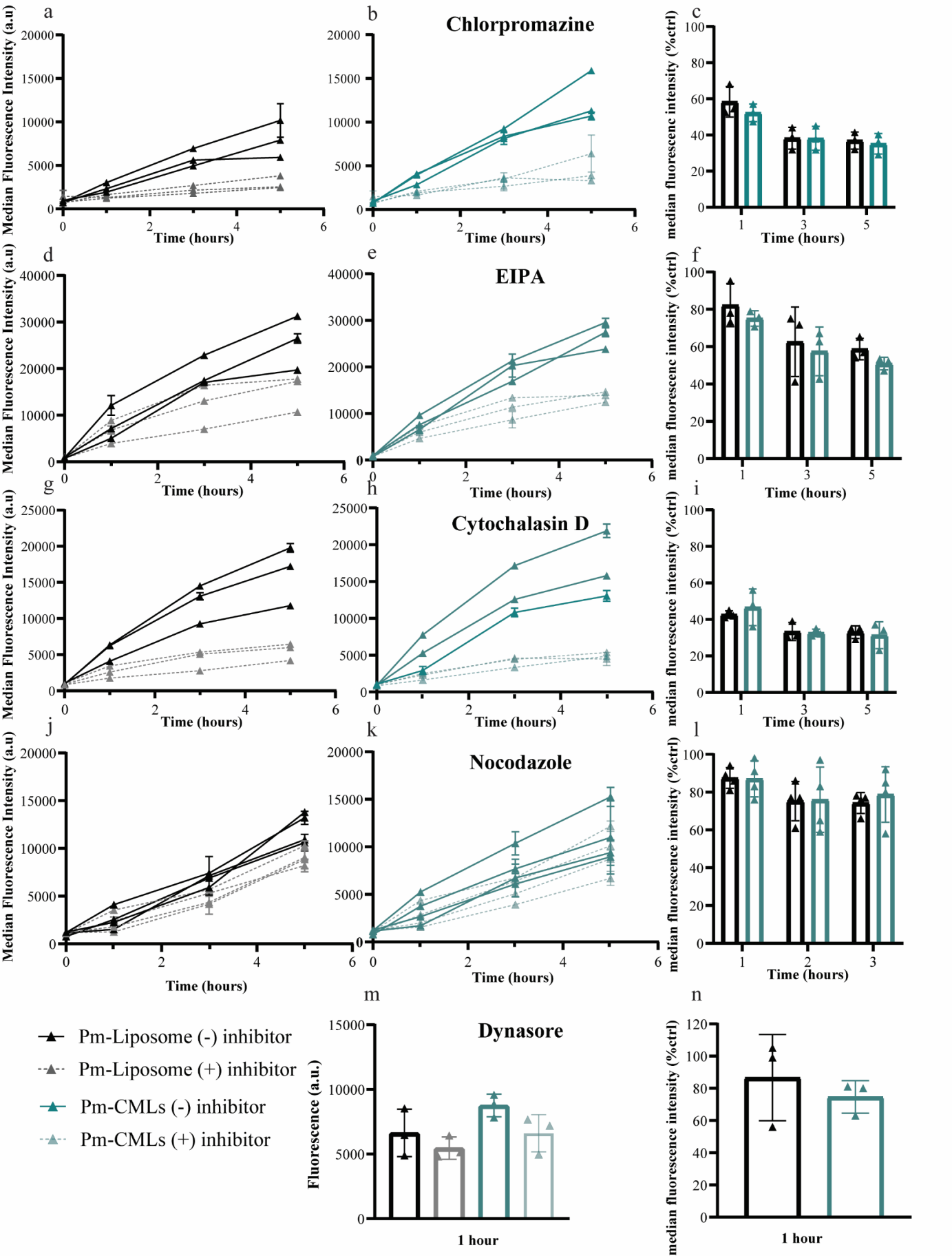


**Figure S4 – Characterization of nanoparticle uptake mechanisms.** A panel of inhibitors was used to determine uptake mechanism of Pm-Liposomes and Pm-CM-Liposomes. Cells were incubated with 20 µg/mL particles for increasing time in standard conditions or in the presence of inhibitors of endocytosis, at the following concentrations: 5-(N-ethyl-N-isopropyl) amiloride (EIPA) (Tocris) 100 μM; Chlorpromazine hydrochloride (Sigma Aldrich) 10 μg/mL; Dynasore (Biovision) 50 μg/mL and Nocodazole (Biovision) 5 μM; Cytochalasin D (Focus biomolecules) 2.5 μg/mL. The average median fluorescence intensity over triplicate samples is shown for 3 independent replicate experiments (4 in the case of nocodazole). In each line, the same data are showed on the right after normalization for the uptake in standard conditions (% of the control).


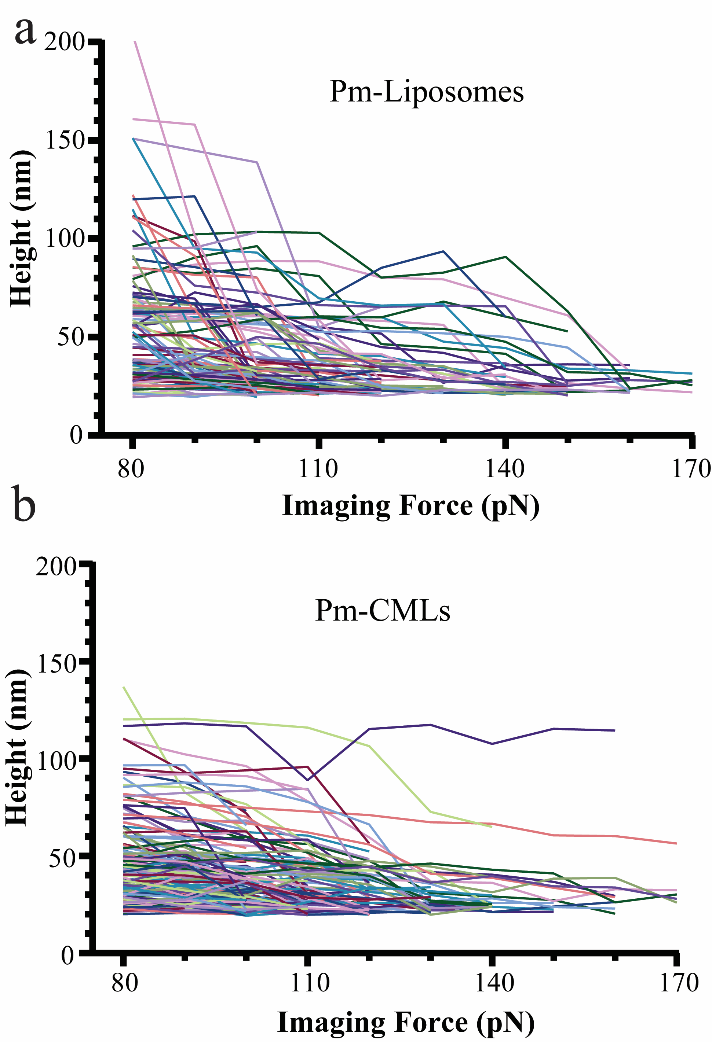


**Figure S5 –Heights of particles measured by AFM at varying imaging force.** a) Height of (a) Pm liposomes and (b) Pm-CM-Liposomes in AFM images acquired at increasing force, as tracked by the script. The corresponding normalized height is shown in Figure 3d.


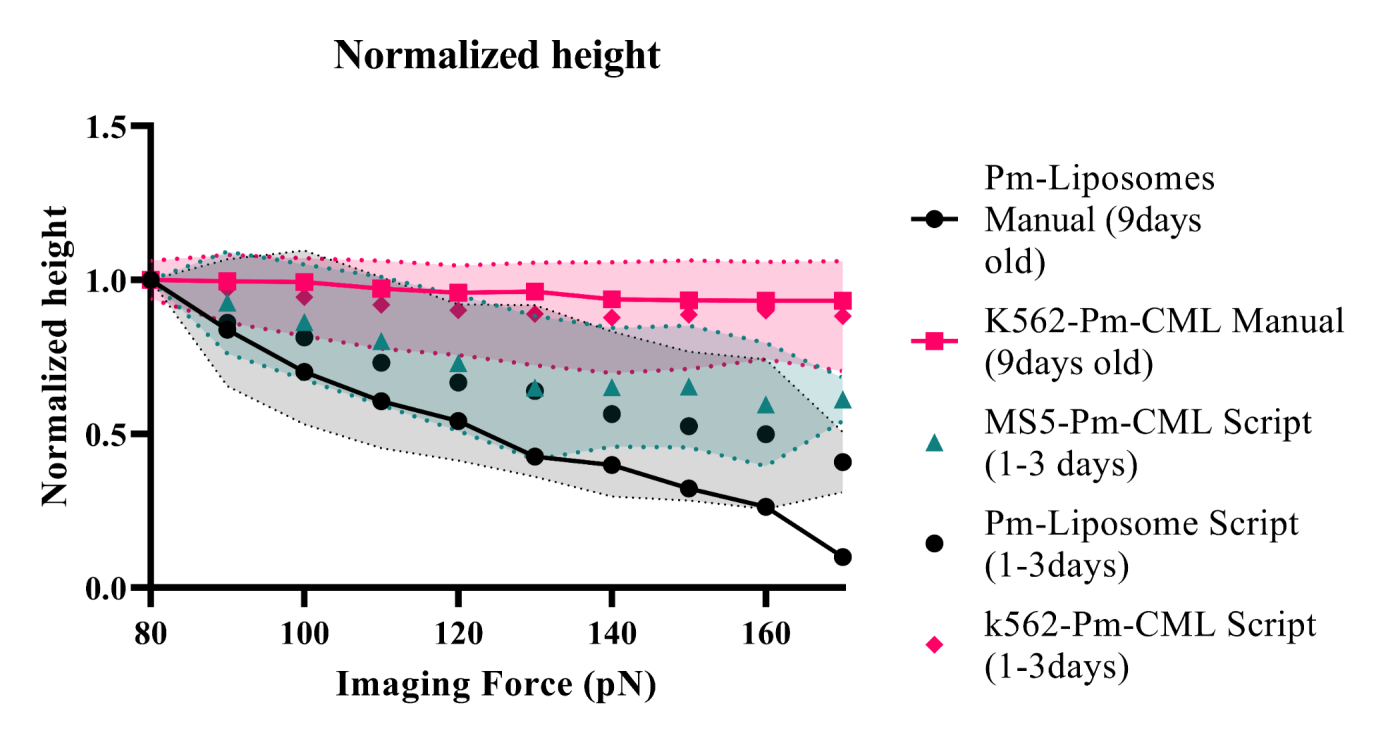


**Figure S6 - Normalized height of K562 and MS5 CM-Liposomes.**  Height of Pm-CM-Liposomes and Pm-Liposomes, with MS5 membrane, or with K562 membrane, obtained by AFM and imaged at various times after preparation (3 days or 9 days after preparation). Heights of Pm-Liposomes and K562-Pm-CMLs were measured by hand in a previous work (here reproduced and shown in dots with a connecting line).^[9]^ The same dataset was used here together with new datasets generated in this work for M5-Pm-CMLs and analysed using a script for image analysis (see Methods for details). Comparable results were obtained when heights were measured manually or using the script developed in this work (normalized average values are shown as dots, coloured area and dotted line indicate SD).


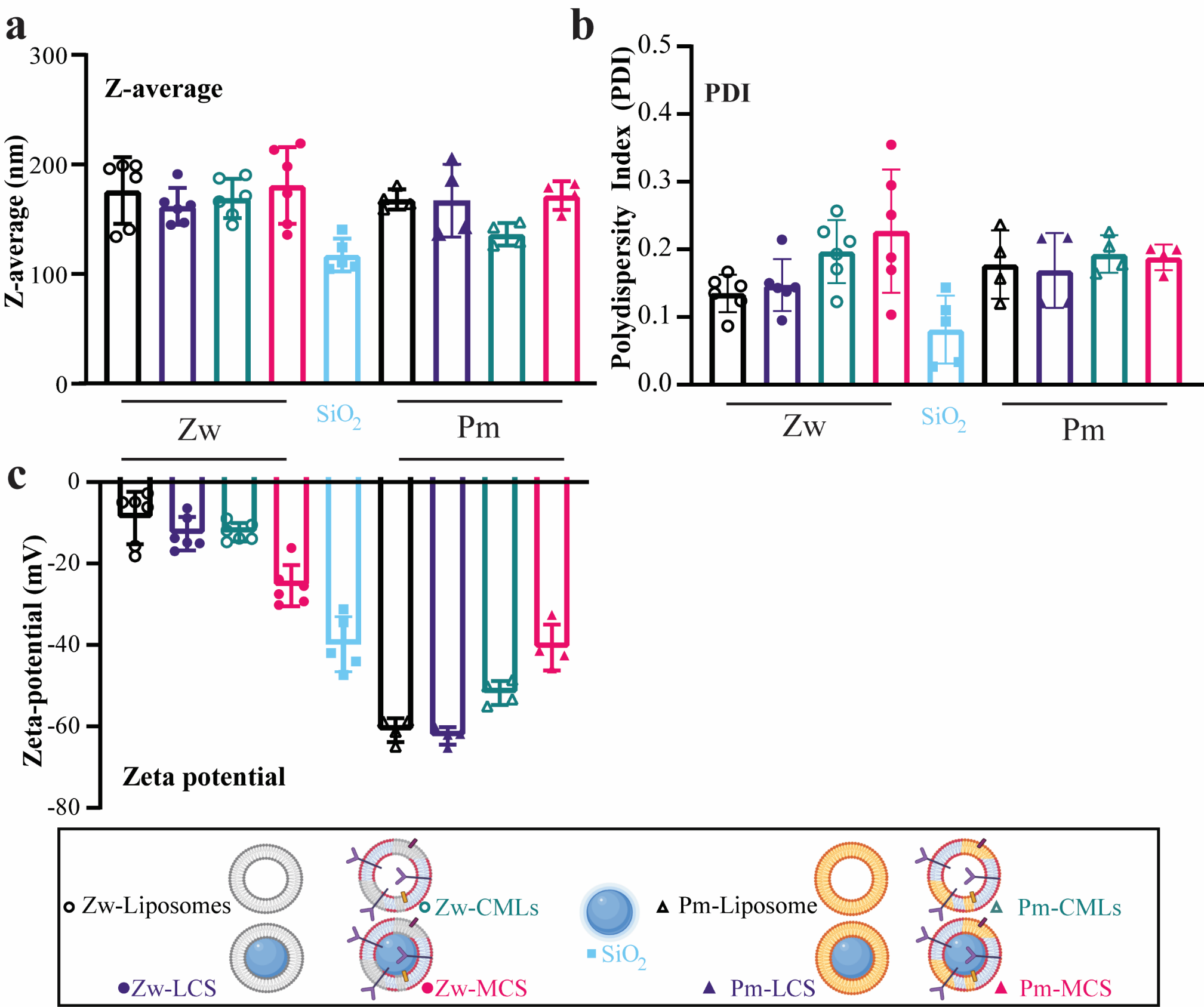


**Figure S7** - a-c) Mean and SD of (a) Z-average diameter and (b) PDI obtained by DLS and (c) zeta potential of multiple batches of particles. The results obtained for multiple batches of both Pm and Zw liposomes and CM-Liposomes before and after deposition on a silica core are shown: each dot represents the average results obtained for a batch measured in triplicate, together with the average and SD of the results obtained for multiple independent batches (Zw: mean ± SD, n = 6 batches), (Pm: mean ± SD, n = 4), (SiO_2_: mean ± SD, n = 4).


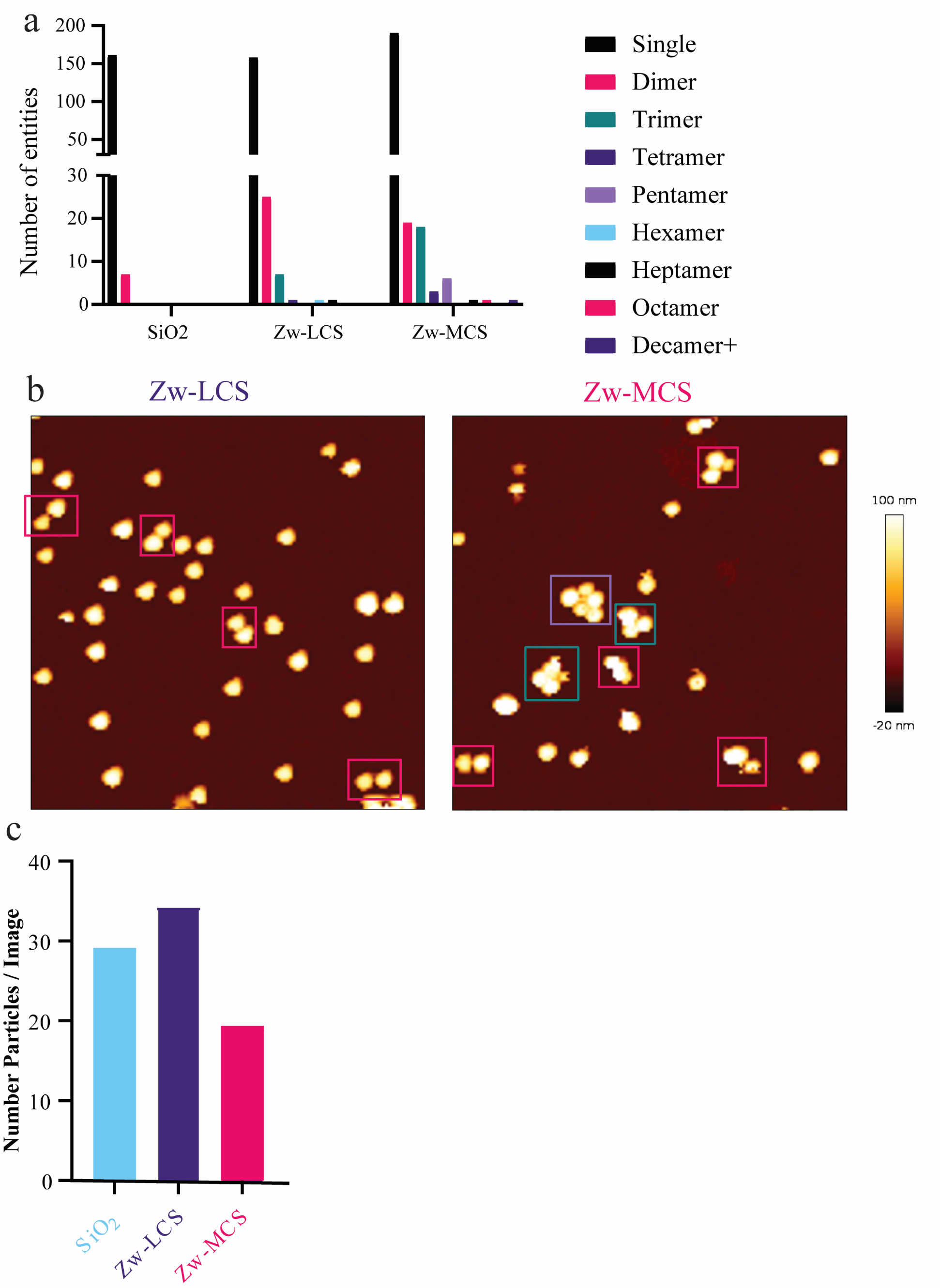


**Figure S8** – **Quantification of clusters.** a) Number of single particles, dimers, trimers, etc. observed in AFM images of bare silica (6 images), Zw-LCS (6 images) and Zw-MCS (9 images). The number of particles and agglomerates was counted manually from the AFM images in Gwydion by creating profile plots to determine the number of spheres in each agglomerate (for details see Methods section). b) Example images for scoring. The width of the images is 3 x 3 µm. c) Total amount of particles counted divided by the number of images taken per sample. Given the lower number of particles per image acquired for Zw-MCS, the chance for dimers and trimers to appear at random is lower.

**
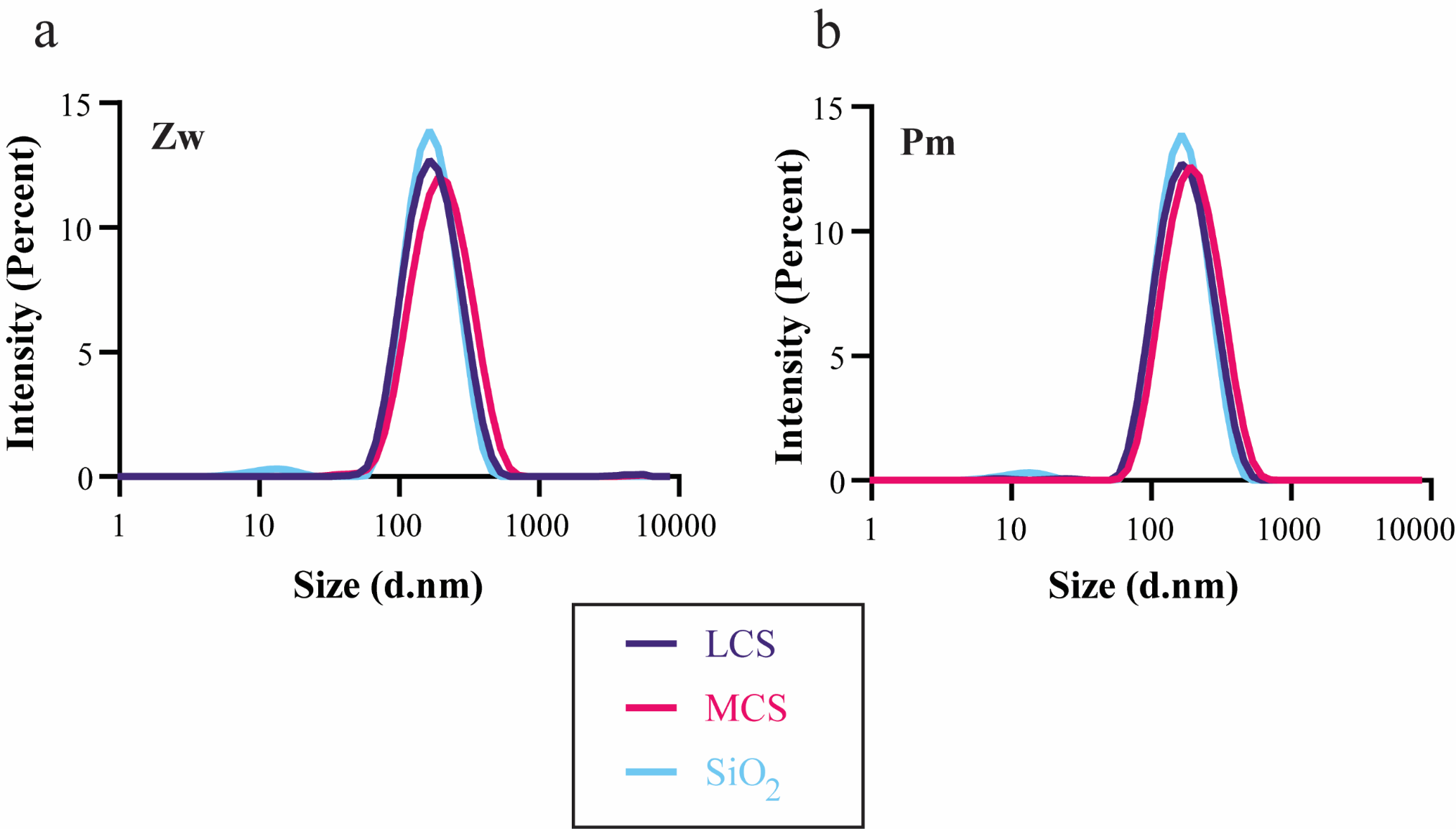
**

**Figure S9 – Stability of membrane coated particles and silica particles in complete medium.** Size distribution of inorganic bare silica particles and (a) Zw-LCS and Zw-MCS, or (b) Pm-LCS and Pm-MCS in cell culture medium supplemented with 10% FBS as measured by DLS. The average intensity of three dispersions measured in triplicate is shown (~100 µg/mL Silica).


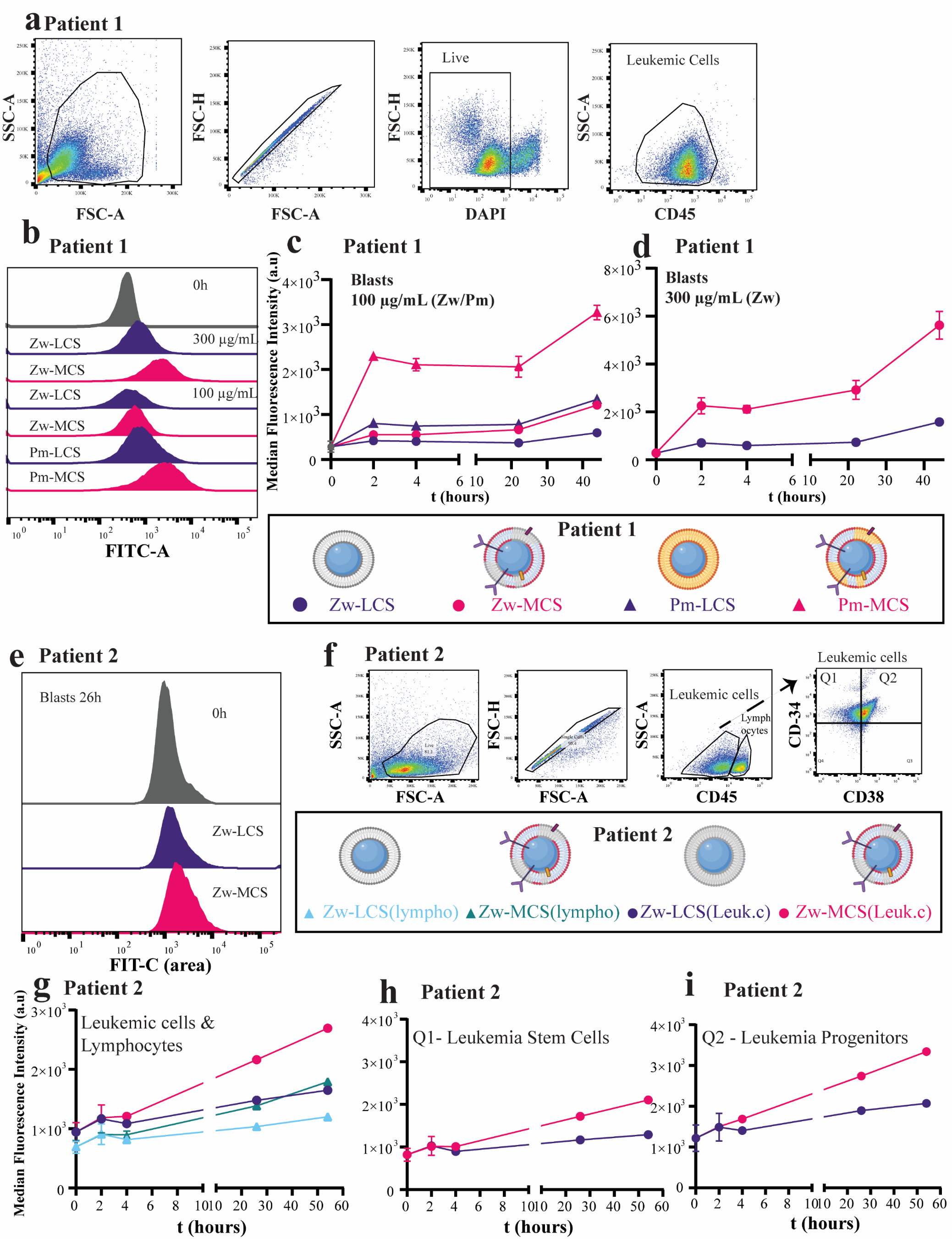


**Figure S10 – Uptake of MCS and LCS in primary patient cells.** Panels a-d are results from Patient 1 in 10% FBS, while panels e-i show results from Patient 2 in 25% FBS. a) Gating strategy to select live leukemia blasts for Patient 1 cells. b) Staggered overlaps of the fluorescence distribution of cells exposed to the different nanoparticles for 2 hours. c) Median fluorescence of primary leukemia blast cells over time, upon incubation with c) 100 µg/mL Zw-LCS, Zw-MCS, Pm-LCS and Pm-MCS, and d) 300 µg/mL Zw-LCS and Zw-MCS. e) Overlap of fluorescence distribution of primary blasts incubated with 200 µg/mL nanoparticles from Patient 2 cells in 25% FBS supplemented medium. f) Gating strategy employed to select live leukemia stem cells (Q1-Leukemia Stem Cells, CD34+, CD38 low) and leukemia progenitor cells (Q2-Leukemia Progenitors, CD34+, CD38 high) for Patient 2 cells in 25% FBS. g) Median fluorescence of primary blasts and lymphocytes incubated with 200 µg/mL Zw-particles for increasing time, and the same for h) gated leukemia stem cells, and i) for leukemia progenitor cells. All experiments were performed in duplicate and the average median cell fluorescence and SD over the two replicate samples are shown. Antibody staining for the gating was performed after exposure (See Methods for details). Figures S10c,d show one experiment with patient cells, where the average and SD of the median cell fluorescence intensity over 2 replicate samples was calculated (mean ± SD, n = 2). Figure S10g-i shows the same for another experiment with cells from a different patient (mean ± SD, n = 2). Figure legends created with Biorender.com.


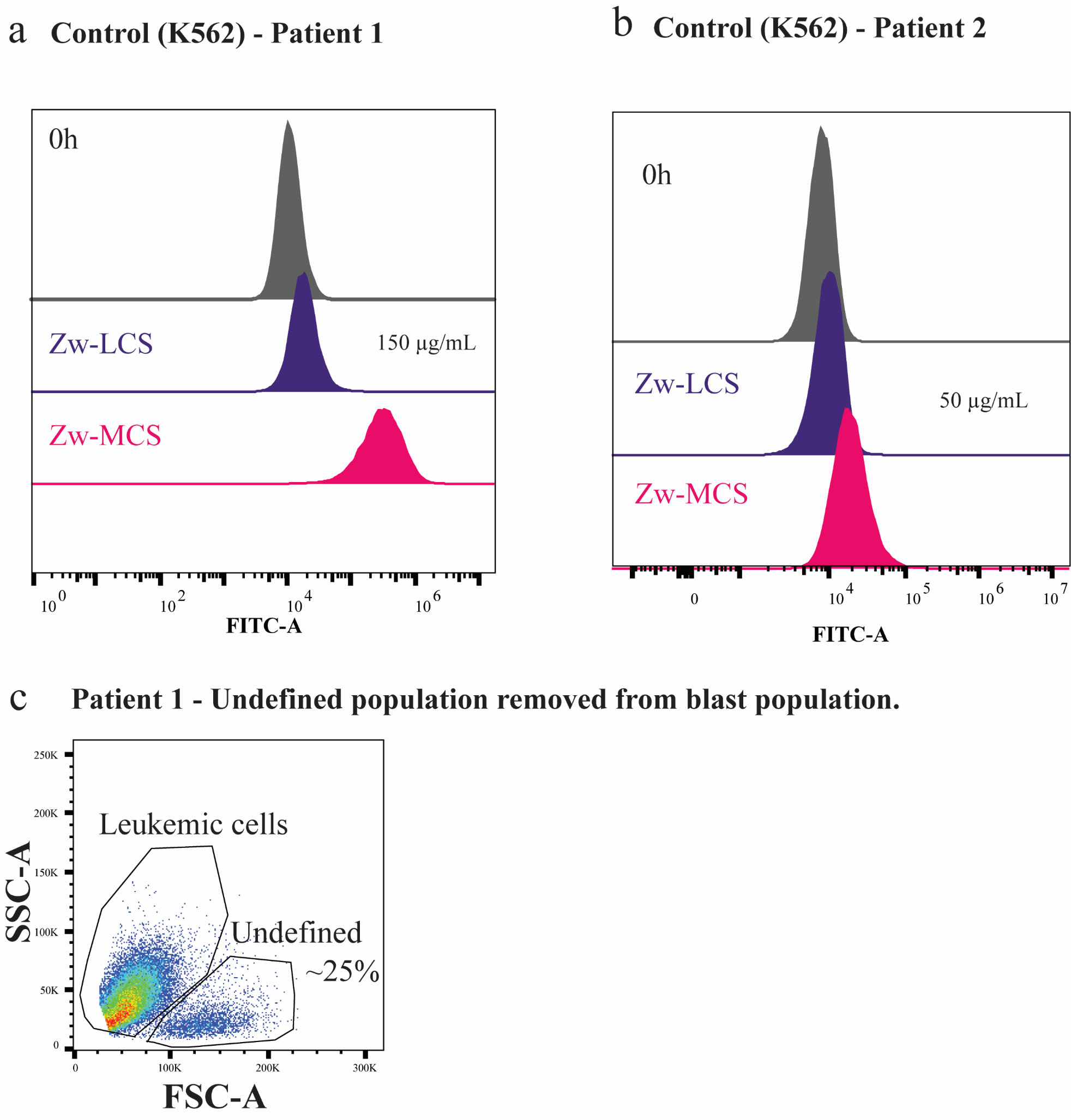


**Figure S11 - Fluorescence of K562 leukemia cells incubated with the batches of particles used in the experiments with primary cells (shown in Figure S10).** a-b) Fluorescence distribution of K562 cells incubated with the same Zw-LCS and Zw-MCS batches used in the experiment for (a) Patient 1 and (b) Patient 2 (50 µg/mL for 6 hours and 150 µg/mL for 3 hours, respectively, in medium supplemented with 10% FBS). Uptake was quantified in K562 as a control to confirm that the membrane nanoparticles used for the experiments with primary cells showed higher uptake than the MCS. c) Side scattering (SSC) versus forward side scattering (FSC) plot of the primary cells of Patient 1, showing the presence of a second undefined population. This subpopulation was excluded from the leukemic cell (blast) population based on scattering.
